# Biological basis and clinical translation prospects of circulating cell-free DNA in precision management of breast cancer (Review)

**DOI:** 10.3892/ol.2026.15570

**Published:** 2026-04-02

**Authors:** Li-Zhuang Fan, Ai-Lan Xu, Ya-Ru Xu, Luo Yang, Fang Yuan, Xin Nie

**Affiliations:** 1 Emergency Department, Shandong Provincial Third Hospital, Tianqiao, Jinan, Shandong 250014, P.R. China; 2 School of Pharmacy, Chengdu University of Traditional Chinese Medicine, Wenjiang, Chengdu, Sichuan 611137, P.R. China; 3 Packaging Design Research Institute, School of Art and Design, Hubei University of Technology, Wuhan, Hubei 430000, P.R. China; 4 Department of Breast Surgery, The First Affiliated Hospital of Shandong First Medical University and Shandong Provincial Qianfoshan Hospital, Shandong Medicine and Health Key Laboratory of General Surgery, Jinan, Shandong 250014, P.R. China; 5 Department of Oncology and Hematology, Dongping County People's Hospital, Dongping, Tai'an, Shandong 271500, P.R. China; 6 Department of Orthopedic Surgery, The First Affiliated Hospital of Shandong First Medical University and Shandong Provincial Qianfoshan Hospital, Shandong Key Laboratory of Rheumatic Disease and Translational Medicine, Jinan, Shandong 250014, P.R. China

**Keywords:** cfDNA, biological characteristics, ddPCR, NGS, breast cancer, early diagnosis and screening, treatment monitoring, prognosis evaluation

## Abstract

Circulating cell-free DNA (cfDNA) has emerged as a valuable non-invasive biomarker that provides unique insights into the spatial and temporal heterogeneity of tumors, serving a key role in cancer precision medicine. Applications of cfDNA include detecting disease progression before clinical and radiological confirmation, identifying actionable genomic alterations, monitoring treatment response, revealing mechanisms of treatment resistance and assessing prognosis. The latest progress in research on the biological characteristics of cfDNA has provided unprecedented molecular insights into cancer diagnosis and treatment, thereby markedly expanding its clinical application scope. The present review explored the latest findings in cfDNA biology and its applications in early diagnosis, treatment monitoring and prognostic assessment of breast cancer. The present review proposed a novel staged transformation strategy guided by the disease progression stage of breast cancer and the maturity of cfDNA technology. The present review aimed to systematically and efficiently promote the application of cfDNA in the full-cycle management of breast cancer. Furthermore, the present review predicted cutting-edge directions such as multi-omics integration, artificial intelligence assistance and ethical considerations. Although cfDNA holds notable promise, its clinical translation is still limited by low-abundance signals in early-stage tumors, insufficient detection standardization and the complexity of bioinformatics interpretation. The present review was based on the integration of existing evidence; however, majority of studies are still retrospective or small-sample prospective, indicating a need to elevate the evidence level. Future research should focus on developing ultra-high-sensitivity multi-omics technology, establishing an international standardization system and verifying the actual effectiveness of cfDNA in guiding clinical decisions through large-scale prospective clinical trials. The ultimate goal is to realize the comprehensive application of cfDNA in early screening and personalized treatment of breast cancer.

## 1. Introduction

According to the 2020 Global Cancer Statistics Report, there were 2.36 million newly diagnosed cases of breast cancer, accounting for 11.7% of the novel cases of cancer in women worldwide. This has elevated breast cancer to the most common malignant tumors among women globally (1). A 2022 study further predicted that breast cancer would continue to rank first in the incidence and mortality of cancer in women, while lung cancer would become the most common cancer type among men (2). Breast cancer imposes a notable economic burden worldwide. The average annual direct medical expenses for patients reaches thousands of dollars across various countries, while indirect costs, such as loss of productivity, lead to huge economic losses reaching billions of dollars (3). It is worth noting that the incidence of breast cancer among young women (particularly those of working age) is on the rise. These patients not only face the dual risks of unemployment and disability, but also bear enormous economic and psychological pressure (4). In addition, breast cancer treatment may lead to chronic pain, mental health problems such as anxiety and depression and changes in body image, which seriously affect the quality of life of patients (5). Although mammography has been extensively adopted, its sensitivity is limited, the false-positive rate is high and it is difficult to reliably detect lesions in fast-growing interphase cancer or dense breast tissue. These limitations highlight the urgent need to improve screening methods (6–8). Simultaneously, biopsy for common metastases such as lung, bone and brain is invasive, technically difficult, expensive and dependent on skilled operators (9,10). In summary, the limitations of existing diagnosis and monitoring methods, as well as the high economic burden, morbidity, mortality and disability rate of breast cancer, jointly highlight the urgent need to develop innovative early detection and treatment technologies. In this context, liquid biopsy technology, particularly circulating cell-free DNA (cfDNA) analysis, as a non-invasive, repeatable and real-time innovative tool to reflect tumor dynamics, is changing the pattern of accurate diagnosis and treatment of tumors at an unprecedented speed.

Targeting its advantages of non-invasiveness, capacity for serial sampling and high patient compliance, cfDNA analysis provides a novel breakthrough for the whole process management of breast cancer. With further understanding of the biological characteristics of cfDNA (such as fragmentation patterns and methylation characteristics) and the growing maturity of next-generation sequencing (NGS) technology, cfDNA analysis can now detect tumor specific mutations, copy number variations (CNVs) and epigenetic changes with high sensitivity. The present review aimed to systematically elaborate the latest progress and transformation path of cfDNA in the precise diagnosis and treatment of breast cancer. In contrast to previous reviews that primarily focused on general technical principles or pan-cancer applications (11,12), the present review focuses on breast cancer specific scenarios, committed to building a complete evidence chain from ‘biomarker traits’ to ‘clinical decision support’. Specifically, the present review aims to: i) Systematically review the frontier analysis dimensions beyond gene mutation, including the emerging applications of cfDNA methylation profiling and fragment omics in early detection, molecular typing and prognosis prediction; ii) integrate and critically evaluate the evidence-based association between key gene mutations [such as PIK3CA, tumor protein p53 (TP53) and ESR1] and clinical endpoints [such as early recurrence, treatment resistance and overall survival (OS)]; and iii) analyze the transformation path and implementation challenge of cfDNA analysis in the dynamic monitoring of therapeutic response and tracking of drug resistance mechanism in advanced breast cancer. Based on the aforementioned focus, the present review aims to provide clinicians and transformation researchers with a reference framework with clear structure, clear evidence classification and clinical operability, thereby promoting the ultimate value of cfDNA analysis in the practice of breast cancer precision medicine.

## 2. Methodology

The present study used a narrative review method to systematically evaluate the biological basis and clinical translation prospects of cfDNA in breast cancer management. To ensure the systematic and representative nature of the evidence, the present review conducted a systematic search in the PubMed database in September 2024, covering the period from 2016 to 2025. The search strategy centered on three major themes: Biological characteristics of cfDNA, detection technology and its application in early diagnosis, treatment monitoring and prognosis assessment of breast cancer, and combined use of subject words and free words to optimize the recall rate. The full search strategies for all databases are provided in Data S1. Literature screening was completed independently by two researchers. First, a preliminary screening was conducted based on titles and abstracts and then the full text of eligible literature was evaluated. Inclusion criteria were as follows: i) Original research or high-quality reviews focusing on human breast cancer; ii) the research content directly involves the clinical application of cfDNA; and iii) it is published in English. The final included literature forms the basis of the present review and supports the preparation of each analysis table in the article (such as Table SI, Table SII, Table SIII, Table IV). These tables focus on the key evidence of cfDNA in pathogenesis, efficacy monitoring and prognostic gene mutations, respectively. Using the aforementioned process, the present review aimed to build a transparent and rigorous evidence system to provide reliable support for the arguments and conclusions proposed.

## 3. Biological characteristics of cfDNA

**Figure 1.**
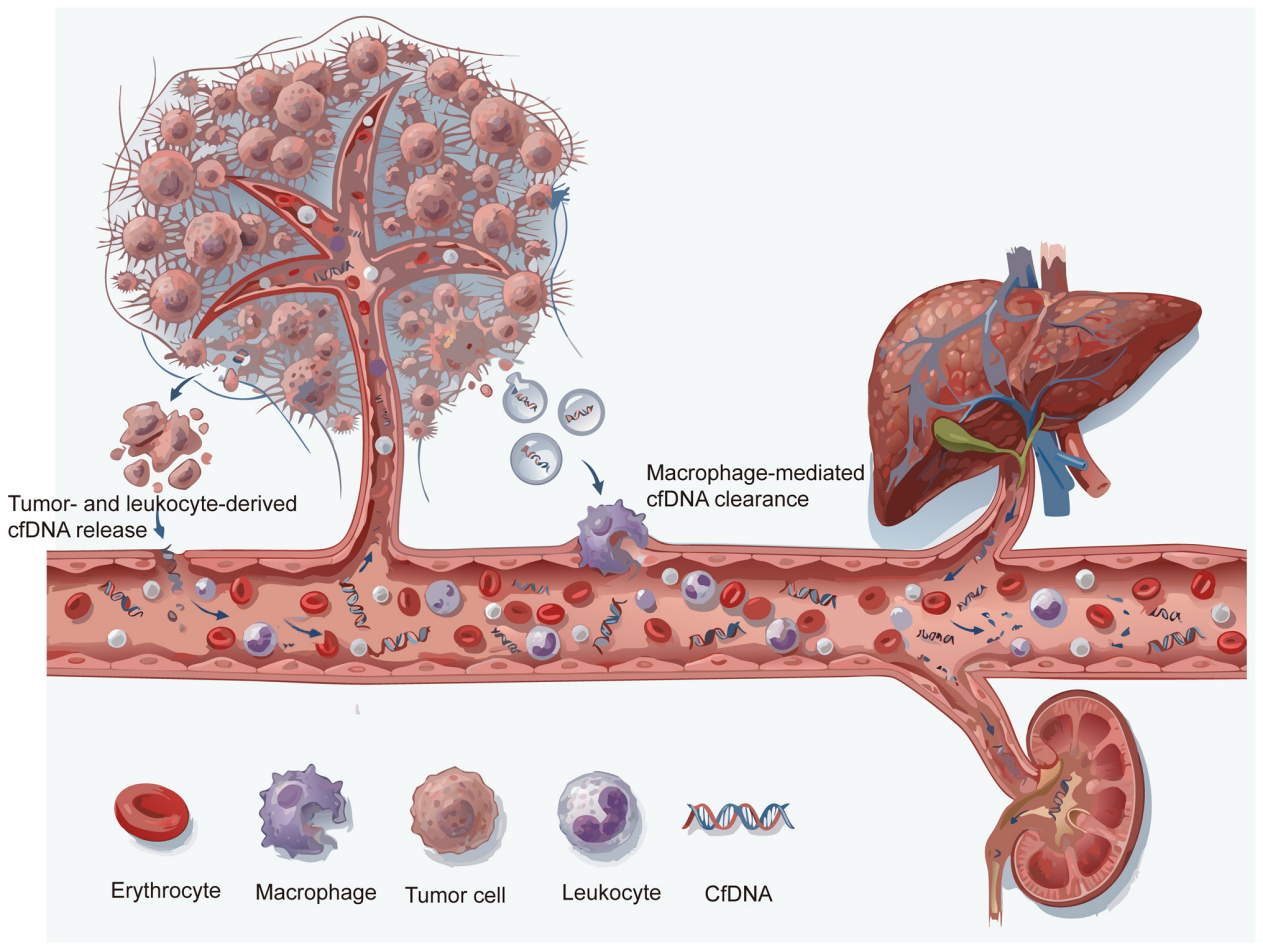
Release and clearance pathways of cfDNA. cfDNA, circulating cell-free DNA.

cfDNA is a key biomolecule that exists in a variety of body fluids and serves a key role in both basic research and clinical applications. cfDNA refers to DNA fragments released from cells into biological fluids such as blood, ascites, pleural effusion and urine (13). In healthy individuals, plasma cfDNA mainly originates from leukocytes (55%), erythrocyte precursor cells (30%), vascular endothelial cells (10%) and hepatocytes (1%). However, in patients with cancer, the proportion of cfDNA released by tumor cells increases markedly. cfNDA is mainly released through extracellular vesicle pathways, such as apoptosis, necrosis and exosomes of tumor cells, and exists in double-stranded and single-stranded forms. It is worth noting that the concentration of cfDNA in serum can reach 3–24-fold higher compared with that in plasma (14–16). Although dying cells release large amounts of DNA, <10% of it can be detected as cfDNA in plasma. Furthermore, the detection levels of cfDNA from different cell sources may differ from the expected ratio by up to a 1,000-fold, typically <0.1% (17). The clearance of cfDNA involves multiple biological pathways and organ systems, including macrophage-mediated phagocytosis, filtration functions of the kidney and liver, plasma nucleases and interactions with other blood components such as serum proteins (e.g., albumin, lipoproteins), divalent cations (Ca^2^+, Mg^2^+) and other circulating nucleic acids. These clearance mechanisms may work synergistically to jointly regulate the levels of cfDNA in circulation, but their specific contributions and proportions still warrant further study to elucidate (18,19). The dynamic fluctuations of cfDNA in the blood result from a complex balance between release and clearance mechanisms. Therefore, the presence and duration of cfDNA in circulation is regulated by the interaction of these processes, highlighting the need to further explore its regulatory mechanisms (Fig. 1).

Recent research revealed that cfDNA fragment length distribution is one of the key features in distinguishing patients with cancer from healthy individuals. The length of cfDNA fragments in plasma samples from cancer patients typically concentrate in the range of 140–180 bp, while those from healthy individuals are generally longer, between 160 and 265 bp (20). In tumor tissues, DNA hypomethylation and high gene expression may promote the generation of shorter cfDNA fragments (21). In addition, the ends of cfDNA molecules derived from nucleosomes are shorter and have unique end sequence characteristics. This implies that by screening cfDNA molecules with specific end characteristics, a higher proportion of tumor-derived cfDNA can be enriched in patients with cancer, thereby improving diagnostic accuracy (22). In the circulation system, cfDNA mainly exists in two forms: Encapsulated in extracellular vesicles (such as apoptotic bodies) or bound to proteins. In tumor-related states, cfDNA-protein complexes have a higher protein-DNA ratio, particularly in pathways associated with ion channels, protein binding, transport and signal transduction (23). Compared with healthy individuals, the cfDNA profiles of patients with cancer exhibit a higher proportion of short fragments and a lower C-terminal preference (24). Concurrently, notable variations in cfDNA fragment length exist among different tumor types. The length variation of cfDNA fragments derived from tumors is markedly higher compared with that of fragments derived from healthy tissues (25,26). Although blood is the most commonly used sample type for cfDNA analysis in clinical practice, other body fluids such as ascites and pleural effusion can provide supplementary diagnostic information that cannot be detected in blood-derived cfDNA. This information is key to understanding the tumor biology and clonal dynamics of primary tumors and metastases (27,28). Furthermore, cfDNA sequencing technology can also reveal clonal hematopoiesis (CH) and notable increases in inflammation levels in the tumor microenvironment (28).

In addition to the field of oncology, cfDNA has identified extensive applications in sepsis diagnosis, type 2 diabetes mellitus management, prenatal screening, cardiovascular disease research and transplantation medicine (29–31). The identification of cfDNA has provided novel avenues for non-invasive diagnosis, enabling dynamic disease monitoring using liquid biopsy. With increasing cfDNA research, multiple subtypes have been identified, including circulating tumor DNA (ctDNA), circulating mitochondrial DNA (cf-mtDNA), cell-free fetal DNA (cf-fDNA), monocyte-derived cfDNA and nucleosome-associated cfDNA. Among these, ctDNA has demonstrated particularly notable application potential in oncology and has become the major focus of current cancer research. Subsequent sections focus on the biological properties and clinical relevance of ctDNA, cf-mtDNA and cf-fDNA.

### Main subtypes of cfDNA

#### ctDNA

ctDNA refers to cfDNA fragments released by tumor cells, accounting for ~0.01% of total cfDNA, with a concentration range in blood samples typically between 5–1,500 ng/ml. The cfDNA/ctDNA ratio is closely associated with tumor burden and proliferation rate (32,33), making it a key biomarker for the assessment of genetic and epigenetic changes in tumors (34). Most ctDNA fragments are >100 bp in length (20) and their concentration changes can reflect tumor burden and treatment effect in real time. Due to its high clinical sensitivity, ctDNA is considered a promising tool in monitoring tumor recurrence (35,36). ctDNA fingerprinting technology improves the specificity and sensitivity of tumor response assessment and can detect tumor recurrence earlier than imaging-based diagnosis (37). However, the extremely low levels of ctDNA in circulations, particularly in early-stage cancer, poses challenges for its extensive clinical application. Developing advanced detection technologies is key to overcoming this limitation.

### cf-mtDNA

cf-mtDNA accounts for a small proportion of the total cfDNA in blood and other body fluids. cf-mtDNA levels are associated with hormone receptor expression and high sensitivity to glucocorticoids can lead to a decrease in cf-mtDNA levels (38,39). cf-mtDNA can serve as a biomarker for metabolic disorders, including type 2 diabetes mellitus, diabetic ketoacidosis, hyperglycemia, hyperosmolar hyperglycemic states, hypoglycemia and gout, and can also be used in the assessment of inflammatory diseases such as sepsis, lung abscess, mastitis and acute appendicitis (40). Furthermore, previous studies have observed that cf-mtDNA levels rapidly increase under acute psychosocial stress (41–43). Plasma cf-mtDNA fragments exhibit a unique size distribution with a peak at ~90 bp (44). Low cf-mtDNA copy number is associated with increased risk of type 2 diabetes mellitus and metabolic syndrome (45).

### cf-fDNA

cf-fDNA mainly originates from the placenta and enters the maternal circulation system. cf-fDNA has been extensively used in non-invasive prenatal testing (NIPT) to screen for chromosomal abnormalities such as trisomy 21 (Down syndrome), trisomy 18 and trisomy 13. cf-fDNA is present as short fragments in maternal plasma (46). Previous research has reported that the peripheral circular DNA molecules of cf-fDNA usually exhibit low methylation levels and are rapidly cleared from the maternal circulation after delivery. Although cf-fDNA is mainly applied in prenatal diagnosis, the extremely short half-life, estimated to be <1 h, is similar to the clearance rate of linear cfDNA, suggesting the general rule of rapid metabolism of cfDNA in the body, which has reference significance in understanding its dynamic changes (47). Selective amplification of cf-fDNA enables targeted analysis in maternal plasma, making it a potential tool for non-invasive prenatal paternity testing (48).

**Figure 2.**
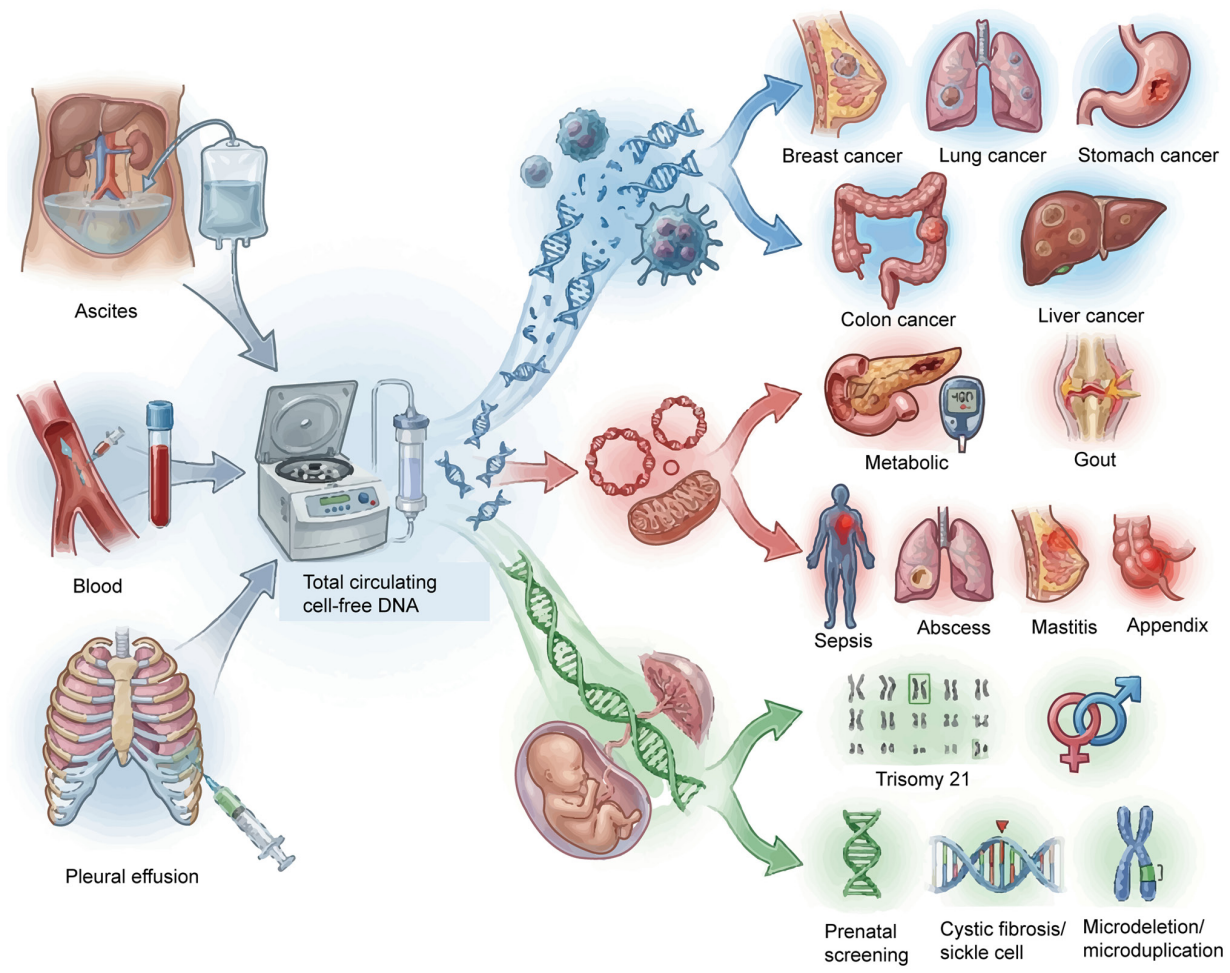
Different samples and cfDNA subtypes. The main sources of cfDNA include clinical samples such as blood, ascites and pleural fluid. Based on its origin and function, cfDNA can be categorized into several subtypes: ctDNA, mainly associated with malignant tumors including breast, lung, stomach and colon cancer; cf-mtDNA, linked to metabolic disorders such as type 2 diabetes mellitus, diabetic ketoacidosis and hyperglycemic hyperosmolar syndrome; and cf-fDNA, primarily used for prenatal screening of fetal chromosomal abnormalities (for example, trisomy 21, Edwards syndrome), fetal sex determination, detection of fetal genetic diseases (for example, cystic fibrosis, sickle cell anemia), and detection of fetal microdeletions or duplications. In addition, cfDNA levels may be elevated in inflammatory conditions such as sepsis, lung abscess, mastitis and suppurative appendicitis. cfDNA, circulating cell-free DNA; ctDNA, circulating tumor DNA; cf-mtDNA, cf-mitochondrial DNA; cf-fDNA, cf-fetal DNA.

Although ctDNA, cf-mtDNA and cf-fDNA have notable potential, their extremely low concentrations in the circulation limit their clinical applications, particularly in the early detection of diseases such as cancer (49,50). This challenge is particularly pronounced in cases with low tumor burden. The continued development of high-sensitivity detection technology is key to promoting its extensive clinical application and ensuring its role in precision medicine (Fig. 2).

### Molecular probes to decode tumor biology

As a carrier of molecular information for tumors, cfDNA provides biological insights into revealing tumor-specific mutations, drug resistance mechanisms, tumor heterogeneity, clonal selection and epigenetic marks. Therefore, cfDNA has become a notable tool in understanding tumor biology and disease progression (12,51). Highly proliferative cancer cells are prone to cell cycle disorders and apoptosis, and apoptosis induced by their rapid proliferation may release cfDNA. This release mechanism makes it possible to detect tumor-related genetic and epigenetic changes, including drug resistance mechanisms, in plasma DNA, providing a non-invasive method in monitoring tumor evolution and treatment response (19).

### Methylation modification and functional interpretation

DNA methylation is a heritable and reversible epigenetic modification that is tightly regulated and highly conserved in normal cells. However, in tumor cells, DNA methylation patterns are markedly altered, including silencing of tumor suppressor genes through hypermethylation, activation of proto-oncogenes through hypomethylation and increased genomic instability. The fragment size and terminal distribution of cfDNA molecules will vary depending on their DNA methylation status. DNA methylation affects cfDNA fragment size by regulating nuclease cleavage preference during apoptosis (52). Lower DNA methylation levels lead to a looser chromatin structure, increasing nucleosome accessibility, thereby enhancing nuclease activity in exposed regions, resulting in shorter DNA fragments (53). Differential methylation analysis of multiple genes in cfDNA has potential application in disease screening. In 2023, Manoochehri *et al* (54) identified differential methylation in cfDNA from independent plasma samples, particularly in differentially methylated regions associated with sperm-associated antigen 6, LINC10606, tubulin folding cofactor D and zinc finger protein 750 genes. By analyzing the methylation signatures of these differentially methylated regions, researchers constructed a methylation score. This score demonstrated notable potential in distinguishing patients with triple-negative breast cancer (TNBC) from healthy controls, suggesting that the methylation score could serve as an effective biomarker for TNBC diagnosis (54). For example, in patients with TNBC, all genes except GATA3 demonstrated markedly higher methylation frequencies compared with that in healthy individuals. In addition, Wnt5A and Sox17 methylation were markedly associated with poor prognosis and shorter survival, while kallikrein-related peptidase 10 methylation was associated with more aggressive clinicopathological features and higher recurrence rates. These findings highlighted the potential of gene-specific methylation patterns as prognostic markers in breast cancer (55). In a 2021 study, Wang *et al* (56) reported that MutL homolog 1 promoter methylation in cfDNA can lead to DNA repair defects, microsatellite instability (MSI) and thus, promote colorectal cancer development. This study highlighted the role of DNA methylation in regulating gene expression and driving tumorigenesis. Abnormal DNA methylation is one of the hallmarks of human cancer, often occurring in the early stages, making it a key biomarker for early detection and diagnosis. For example, in 2023, Kim *et al* (57) demonstrated that combining the cfDNA methylation markers ring finger protein 135 and lactate dehydrogenase B with α-fetoprotein can identify ~70% of hepatocellular carcinoma cases, markedly improving the liver cancer diagnosis rate. Furthermore, cfDNA methylation analysis can reveal tumor heterogeneity. In February 2024, Heeke *et al* (58) confirmed that the tumor phenotype of small cell lung cancer evolves with disease progression and cfDNA methylation can identify different subtypes at the transcriptional level. This finding provides a valuable tool in guiding personalized treatment strategies. The role of DNA methylation in tumors is not limited to gene regulation and tumor initiation, but also includes identifying tumor heterogeneity, affecting genome stability and helping tumor cells adapt to the environment. Further understanding of these aspects is key to advancing cancer biology research and improving treatments.

### Evolution of tumor drug resistance

cfDNA not only reflects methylation characteristics but also provides key clues to study acquired drug resistance. Tumor drug resistance can emerge at any stage of cancer treatment and is often driven by target gene mutations, drug target amplification and adaptive changes in tumor cells at the genetic, epigenetic and microenvironmental levels. Genetic alterations in DNA repair pathways associated with acquired resistance can be identified using cfDNA analysis. For example, cfDNA sequencing has revealed complementing mutations in BRCA1 and BRCA2 in breast cancer, elucidating potential mechanisms of resistance to platinum-based chemotherapy drugs such as cisplatin and carboplatin (40,59). These complementing mutations can partially or completely restore the DNA repair function of tumor cells, allowing them to effectively repair DNA double-strand breaks induced by platinum drugs, ultimately leading to chemotherapy resistance. Tumor cells can also become resistant to immunotherapy through mutations that promote immune evasion, which can be detected by ctDNA sequencing. In other tumor types, cfDNA has also made notable progress in revealing the tumor microenvironment and drug resistance mechanisms. For example, in 2024, Skoulidis *et al* (60) reported that serine/threonine kinase 11 and Kelch-like ECH-associated protein 1 mutations in lung cancer lead to immunosuppression in the tumor microenvironment, which is characterized by CD8^+^ T-cell dysfunction and an increase in myeloid-derived suppressor cells. These findings in lung cancer suggested that cfDNA analysis has the potential to assess the tumor immune microenvironment and predict the efficacy of immunotherapy, which also has implications for breast cancer. In response to the aforementioned immunosuppressive mechanism, dual immune checkpoint inhibition can restore CD4^+^ T-cell function and reprogram myeloid cells to target the elimination of tumor cells (60). This strategy could help overcome drug resistance and enhance the efficacy of tumor immunotherapy. Identifying immune evasion mechanisms is key to combating resistance to immune-based therapies. In hormone-driven cancer, such as estrogen receptor-positive (ER^+^) breast cancer, endocrine therapy resistance is often associated with mutations or downregulation of ER genes. Estrogen promotes tumor cell proliferation by binding to ER, causing breast gland mitosis. Therefore, patients with ER^+^ breast cancer usually have a lower risk of early recurrence and improved response to endocrine therapy (61). However, several studies have reported that circular RNA (circRNA)-Scm-like with four mbt domains 2 (SFMBT2) can promote the expression of Quaking I protein, which may contribute to endocrine resistance (62). Understanding cfDNA-based resistance mechanisms is key to guiding precision oncology strategies, improving treatment efficacy and developing novel therapeutic interventions. Further studies have demonstrated that increased expression levels of circRNA-SFMBT2 promote the growth of ER^+^ breast cancer cells and exacerbate resistance to endocrine therapies such as tamoxifen (62). ctDNA detection provides a novel approach in studying the resistance mechanism of endocrine drugs (63). As tumors accumulate genetic mutations, cfDNA analysis can track mutations associated with drug resistance (64). cfDNA analysis can detect secondary mutations in key targeted genes [for example, G protein-coupled receptor 155 (GPR155), ADAM metallopeptidase with thrombospondin type 1 motif 20 (ADAMTS20), titin (TTN) and BRAF], thereby promptly identifying the occurrence of drug resistance and optimizing treatment strategies. For example, in 2022, Lee *et al* (65) used cfDNA whole-exome sequencing (WES) to identify secondary mutations, including GPR155 (I357S), ADAMTS20 (S1597P) and TTN (R7H), in patients with lung adenocarcinoma and colorectal cancer following EGFR targeted therapy and chemotherapy. These mutations occur frequently during acquired resistance, highlighting the value of cfDNA sequencing in detecting resistance-associated genetic variants. In breast cancer, ESR1 gene mutations can change the structure or function of the ER, making cancer cells independent of exogenous estrogen. The most common cause of resistance to aromatase inhibitors in patients with breast cancer is ESR1 mutation, which is associated with disease recurrence, progression and resistance to endocrine therapy in advanced breast cancer. Continuous cfDNA monitoring for ESR1 mutations serves a key role in guiding personalized treatment decisions and adjusting therapeutic strategies (66,67). Furthermore, in a 2023 study, Serrano *et al* (68) demonstrated that KIT and platelet derived growth factor receptor α gene mutations in patients with gastrointestinal stromal tumors can be identified using ctDNA sequencing technology. The detection of these mutations is particularly valuable in the context of resistance to targeted therapy, as it is directly associated with patient prognosis. During tumor treatment, novel mutations or gene amplifications may appear that alter drug targets or reduce drug binding efficiency, ultimately leading to drug resistance. In summary, cfDNA serves as a dynamic biomarker that reflects the genetic evolution and drug resistance mechanism of tumor cells throughout the treatment process. By analyzing key genetic changes in cfDNA, clinicians can potentially predict drug resistance and adjust treatment options in a timely manner, thereby improving patient outcomes in the future.

### Liquid capture of early tumor signals

Obtaining tumor genomic information using non-invasive cfDNA analysis enables early detection, treatment monitoring and drug resistance assessment of cancer. CfDNA fragments exhibit unique characteristics based on their source cells, making them important biomarkers for tumor analysis. In 2024, Stanley *et al* (69) analyzed cfDNA fragment patterns and identified that these patterns could reflect the unique biological characteristics of tumors. The study distinguished various cancer types, such as colorectal and early breast cancer andesophageal and gastric cancer, providing a key molecular basis for early tumor detection and screening (69–71). Furthermore, whole-genome deep sequencing of cfDNA has identified a large number of somatic mutations in plasma samples with low tumor burden and has helped to distinguish tumor-derived mutations from those associated with CH in normal germline tissue (72). This finding enables early detection of tumor-related gene mutations and structural variations even when the tumor burden is extremely low, opening up a novel way for more precise cancer diagnosis and treatment. Furthermore, shallow whole-genome sequencing (WGS) of cfDNA further advances clinical monitoring of cancer treatments by accurately and consistently quantifying tumor markers in advanced cancer (73). The detection and analysis of cfDNA provides a solid molecular basis for early tumor diagnosis, prognosis assessment and personalized treatment strategies in the future.

### A notable tool for dynamic monitoring of tumors and non-tumor diseases

cfDNA application extends beyond early detection and encompasses broad dynamic monitoring of therapeutic response assessment, recurrence warning and non-tumor disease monitoring. Surveillance methods not only improve the specificity and sensitivity of tumor detection but also identify tumor recurrence earlier compared with imaging-based diagnosis. Using dynamic and continuous monitoring of cfDNA, changes in tumor genome can be tracked in real time, providing key support for personalized treatment strategies and efficacy evaluation. For example, a previous study by Tie *et al* (74) in 2021 reported that among patients who underwent resection of colorectal liver metastases, those with detectable ctDNA had lower recurrence-free survival (RFS) and OS compared with those without detectable ctDNA. These findings highlighted the prognostic value of cfDNA in early identification of high-risk patients and guiding treatment adjustments. cfDNA WGS technology has demonstrated high sensitivity and reliability in monitoring tumor mutations and treatment response. A previous study by Liu *et al* (75) in 2024 demonstrated that using ultra-low coverage genome sequencing, cfDNA can detect the risk of second primary malignant tumors in patients with Li-Fraumeni syndrome, providing novel biomarkers for early intervention. Similarly, in advanced breast cancer, ESR1 and PI3K mutations detected in plasma ctDNA are markedly associated with prognosis and newly identified mutations can affect subsequent treatment decisions (76). The development of NGS technology has made cfDNA a notable tool for longitudinal monitoring of metastatic breast cancer (77). The value of dynamic cfDNA monitoring not only demonstrates application potential in oncology, but also in non-tumor diseases. In type 2 diabetes mellitus management, cfDNA levels are closely associated with glycemic control and can serve as a key indicator to monitor disease progression and complications (78). In organ transplantation, cfDNA levels associate with transplant prognosis. A previous study by the Moeller *et al* (79) in 2024 reported that increased plasma cfDNA levels after heart transplantation are closely associated with transplant rejection. Monitoring cfDNA levels can help detect rejection early, guide timely clinical intervention and improve patient prognosis in the future. In addition, cfDNA has also exhibited notable monitoring value in kidney and liver transplantation (80,81).

In summary, cfDNA has demonstrated unique advantages in monitoring both tumor and non-tumor diseases, particularly in early diagnosis, disease progression tracking and treatment response assessment. With the continuous advancement of technology, cfDNA has the potential to develop into a routine diagnostic and monitoring tool in clinical practice. This progress holds promise not only in enhancing treatment outcomes but also extending patient survival and improving the quality of life.

## 4. Cutting-edge technology and methodological innovation in cfDNA detection

With the continuous development of biotechnology, the extraction and analysis of cfDNA have become increasingly efficient and convenient. Real-time monitoring of diseases, such as breast cancer and myocardial infarction (82,83), using simple blood samples not only markedly reduces patient trauma and discomfort but also helps optimize the allocation of medical resources and reduce overall medical costs (84). Currently, a variety of cfDNA extraction and analysis technologies have been extensively used in clinical diagnosis, physiological status assessment and basic research. The main detection technologies include droplet digital PCR (ddPCR), NGS and other cutting-edge emerging technologies (85,86). The continuous improvement of these methods has improved the sensitivity and accuracy of cfDNA analysis, further expanding its application potential in precision medicine and disease monitoring.

### ddPCR

The basic principle of ddPCR involves partitioning the sample DNA into a large number of micro-reaction units and achieving absolute quantification of target DNA molecules by counting the number of positive droplets after PCR amplification. The workflow of ddPCR mainly includes key steps such as sample preparation, droplet generation, PCR amplification, data analysis and result reporting. Compared with traditional quantitative PCR (qPCR), ddPCR enables high-sensitivity absolute quantification without the need for a standard curve. The limit of detection in ddPCR reaches 0.01–1.0% of genomic material, demonstrating notably enhanced performance in detecting low-abundance point mutations and CNVs. This inherently high sensitivity and precision make ddPCR indispensable in clinical diagnostics and research applications (87).

One of the key advantages of ddPCR is its high sensitivity and specificity in cfDNA detection. Due to the short half-life of cfDNA, timely and efficient purification is key to ensuring the accuracy of analytical results (88). ddPCR enables precise quantification of cfDNA in plasma and can effectively detect mtDNA after DNA extraction. However, when using plasma directly as a template, the detection process may be affected by vesicular structures such as exosomes (89). In order to improve the efficiency of DNA purification, researchers have increased the cfDNA purification efficiency by up to 95-fold compared with the original method by optimizing digestion conditions, liquid handling parameters and magnetic bead processing procedures (90). Another major advantage of ddPCR is its strong anti-interference ability. Traditional PCR methods are often interfered by inhibitors present in complex biological samples; however, ddPCR can markedly reduce such interference due to its compartmentalized reaction unit, thereby improving the reliability, stability and reproducibility of clinical sample analysis (91). Furthermore, ddPCR has demonstrated notably increased sensitivity compared with traditional qPCR in detecting tumor-associated cfDNA and can still accurately identify genetic variants even when the variant allele frequency is as low as 0.1% (92,93). Previous studies have reported that ddPCR detection of ctDNA has high sensitivity, specificity and accuracy. For example, it has been proven to be an effective tool in the assessment of minimal residual disease in Ewing sarcoma and holds promise in guiding future treatment strategies (94). This finding provides key reference for cfDNA detection of minimal residual disease in breast cancer. Similarly, selecting the appropriate sample type is key to optimizing cfDNA detection. Compared with serum, plasma can reduce the dilution effect of non-tumor DNA, thereby improving the sensitivity of ctDNA detection (95).

However, the automation of ddPCR technology has been constrained by the complexity of liquid handling, particularly during the conversion of ml-scale samples into nl droplets. To overcome this challenge, researchers have developed a novel integrated ddPCR microdevice, realizing a fully automated ‘sample-to-result’ detection process. The device can identify rare tumor mutations with a detection sensitivity as low as ~1% and requires only 2 ml of human plasma sample (96). This innovation provides a practical solution for the automation of liquid biopsies. Further research conducted a systematic evaluation of 15 different cfDNA extraction and downstream analysis technologies (97,98). The results demonstrated that automated extraction protocols and PCR-based quantitative methods performed most notably in terms of consistency and accuracy, with PCR-based methods demonstrating a clear advantage. This indicated the applicability of cfDNA for nucleic acid extraction and mutation detection and highlighted the key role of PCR-based methods in establishing clinically feasible workflows prior to cfDNA analysis (99).

### NGS

NGS has been extensively applied in oncological research and clinical practice. The main sequencing strategies include WGS, WES, transcriptome sequencing (RNA-seq), genome-wide association studies, targeted sequencing, CNVs analysis and MSI detection (100–102). The standard workflow of NGS includes several key steps: Sample preparation, nucleic acid extraction, library construction, sequencing execution and comprehensive data analysis. These steps ensure the accuracy and reliability of genomic information, facilitating early detection of cancer, treatment monitoring and the development of personalized treatment strategies. Sample preparation is the first key step in NGS and its quality directly affects data quality and reliability. A previous study by Ritter *et al* (103) demonstrated that cfDNA was successfully extracted from serum samples stored for 30 years and 24 cancer-specific mutations were identified in 25 sequenced samples from 52 patients with breast cancer. This study highlighted the robustness and specificity of NGS technology, confirming its suitability for the analysis of long-term stored serum samples. Similarly, a previous study of Jiang *et al* (104) in 2020 also reported that cfDNA can maintain high quality and be suitable for NGS even after cryopreservation at −80°C for 1–6 years. After DNA extraction and quality assessment, library construction is a key step in the NGS process, which directly affects sequencing accuracy and data integrity. Using Bioanalyzer and high-sensitivity DNA chromatin immunoprecipitation to determine the concentration and size distribution of cfDNA can comprehensively evaluate the quantity, quality and potential genomic DNA contamination of DNA. This step is key to optimizing the library preparation process and ensure high-quality sequencing results (105).

The traditional method of double stranded DNA library preparation mainly focuses on the analysis of 167 bp double stranded single nucleosomes and other oligonucleosomes derived from cfDNA. However, Troll *et al* (106) innovatively developed an efficient single stranded library preparation technology. This method can construct complex libraries from as little as 1 ng of input DNA in only 2.5 h, while completely retaining the natural ends of template molecules. This single stranded library method not only markedly simplifies the preparation process, but also notably expands the potential application of cfDNA analysis. NGS serves a key role in both cancer research and clinical applications. By continuously optimizing the NGS workflow, clinical needs of patients can be further met, such as faster turnaround times for timely treatment decisions and enhanced sensitivity for detecting low-frequency mutations in early-stage or minimal residual disease settings. Targeted NGS of cfDNA enables dynamic monitoring of tumor heterogeneity and its response to targeted therapies (107). This technology aids in detecting gene mutations in ctDNA, providing valuable information in understanding tumor evolution and precise treatment effect. In tumor genome analysis, a key challenge faced by NGS is to accurately distinguish tumor specific variants from sequencing artifacts and normal germline variants. To address the problem of false-positives, researchers have developed a machine learning model that integrates additional filtering steps. When applied to QIAseq^®^ data (Qiagen GmbH), its sensitivity is 35% and accuracy is 36%, highlighting its effectiveness in capturing tumor specific variation. By integrating germline DNA analysis, the study validated the somatic origin of the identified variants and lastly detected seven variants, six of which were consistent with the germline validation strategy (108).

### Emerging strategies and cutting-edge exploration of cfDNA detection

In recent years, in addition to digital PCR and NGS, a series of emerging detection technologies have also notably enhanced cfDNA analysis capabilities. Traditional cfDNA extraction methods often demonstrate low efficiency and poor purity. To address this, Jeon *et al* (109) developed a nanostructured conductive polymer platform for efficient capture and release of cfDNA. They validated the effectiveness of this platform using unprocessed plasma samples from patients with breast and lung cancer. Research results reported that this platform could recover tumor-specific cfDNA with high yield and purity by improving efficiency. In addition, Raman spectroscopy technology has been explored for cfDNA analysis. Researchers reported notable differences in cfDNA spectral patterns in patients with breast cancer receiving neoadjuvant therapy, suggesting that cfDNA molecular signatures may be associated with disease status (110,111). Similarly, by analyzing cfDNA, researchers have also identified unique biomolecular fingerprints that can distinguish healthy individuals from those with prediabetes and those with type 2 diabetes mellitus (112). In metastatic breast cancer, molecular barcoding technology (MB-NGS) is used to detect ESR1 mutations in cfDNA. Previous studies have demonstrated that MB-NGS has higher sensitivity compared with traditional NGS, enabling the identification of more mutations in cfDNA samples (113,114).

**Figure 3.**
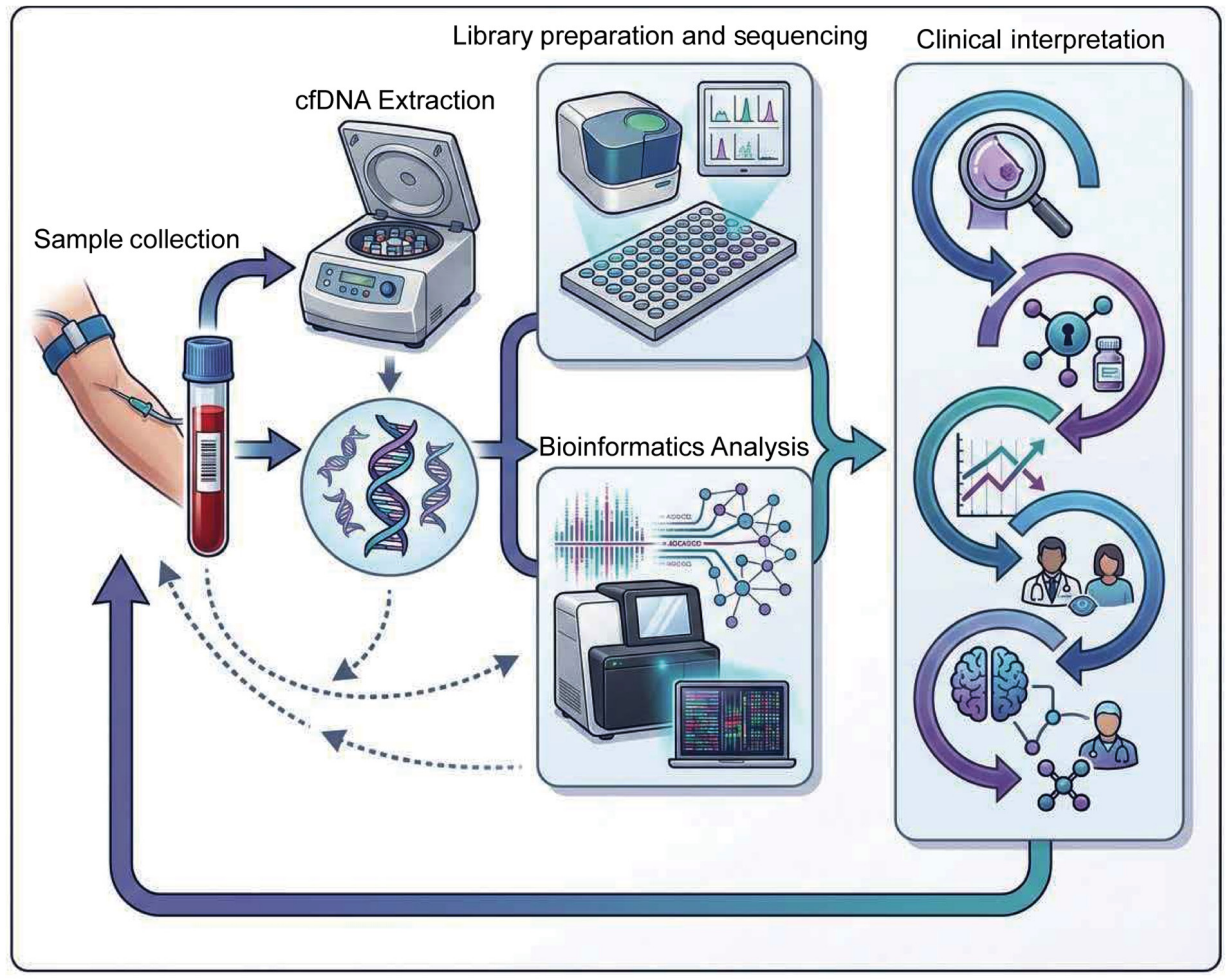
Schematic diagram of the circulating cell-free DNA analysis process presenting a panoramic view of the technical pillars and their transformation into clinical applications.

In summary, the continued progress of these cfDNA analysis technologies has jointly provided strong technical support for early detection, precise diagnosis and personalized treatment of diseases, indicating a broad and promising future in clinical application (Fig. 3).

## 5. Translational prospect of integrating cfDNA into clinical decision-making for the full management of breast cancer

Researchers hypothesize that cfDNA has notable potential in the clinical management of breast cancer if the following application scenarios can be realized: i) Distinguish breast cancer patients from benign breast lesions or healthy individuals to achieve early detection and diagnosis (115); ii) identify and monitor micro-occult metastases to improve the sensitivity of metastasis detection (116); iii) predict OS based on cfDNA analysis to improve the accuracy of prognostic assessment (117); iv) evaluate treatment response using dynamic cfDNA monitoring before imaging evaluation, providing real-time insight into efficacy (118); and v) detect genomic changes associated with treatment resistance to guide the formulation of personalized treatment strategies (119). The successful implementation of these applications may markedly improve the diagnosis, treatment and prognosis management of breast cancer, therefore improving patient outcomes in the future (120).

### Application of early screening and diagnosis

The concentration of cfDNA, modifications (such as methylation, nucleosome positioning and histone modifications) and genetic mutations can all reflect the presence and progression of tumors (121,122). Compared with imaging-based screening and early diagnosis, cfDNA analysis may provide a more convenient and cost-effective method, particularly for patients with occult breast cancer. Traditionally, pathological biopsy is considered the gold standard for breast cancer diagnosis, but it requires imaging guidance and is an invasive procedure. As a non-invasive alternative, cfDNA analysis can effectively meet these diagnostic needs. Early detection of breast cancer markedly affects treatment options and adjuvant therapy decisions, increasing the likelihood of treatment success. Breast cancer cells, particularly aggressive subtypes, exhibit higher mitotic and glycolytic activities compared with normal cells and less aggressive tumor subtypes, resulting in increased cfDNA release and higher ctDNA proportions. This biological characteristic provides a theoretical basis for the application of cfDNA in the screening of aggressive breast cancer subtypes that are rapidly growing, highly invasive and have high mortality (123).

Elevated serum cfDNA levels are closely associated with breast cancer. Variant allele frequencies (VAF) as low as 0.08% can be detected in plasma cfDNA samples from healthy individuals. These mutations are consistent with pathogenic mutations that cause certain individuals to develop benign tumors or invasive breast cancer within a decade (124,125). Furthermore, changes in DNA methylation profiles in blood can be observed several years before clinical detection of breast cancer, suggesting its potential as an early biomarker (126). In patients with breast cancer, cfDNA fragments from hypomethylated regions were shorter compared with that in healthy individuals. In addition, the proportion of short cfDNA fragments in hypomethylated regions is higher compared with that in hypermethylated regions (127). Since cfDNA is enriched in hypomethylated genomic regions, single-base resolution analysis of genome-wide DNA methylation in blood can effectively distinguish early breast cancer from benign tumors (128). Analysis of cfDNA in healthy individuals may become a promising tool for breast cancer screening and early diagnosis, capable of detecting genomic instability and providing key insights into early events in tumor formation.

Nucleosomes serve a key role in cancer by affecting genome structure and gene expression. CfDNA originates from genome regions protected by nucleosomes from enzymatic digestion, with the majority of cfDNA fragments consisting of mononucleosomal DNA. Longer cfDNA fragments, such as dinucleosomes and trinucleosomes, often carry more mutational signatures and may contain additional nucleosomes, thereby providing further insights into tumor biology (129). cfDNA nucleosome profiling analysis can accurately reflect the tissue origin and transcription factor activity of different breast lesions, effectively distinguishing benign and malignant cases (130). Of note, the dynamic changes in transcription factor-associated nucleosomes in plasma cfDNA reveal ER-driven status in breast cancer. The degree of enrichment of transcription factor footprints in plasma samples corresponds to the binding strength of transcription factors in primary tumor tissue, making it possible to identify ER^+^ breast cancer using plasma-based transcription factor footprint analysis (131). Further understanding of these nucleosome-related changes will help elucidate the molecular pathological mechanisms of breast cancer and accelerate the development of more precise cfDNA diagnostic strategies.

### Application of retrotransposon [Alu and long interspersed nuclear element-1 (LINE-1)] CNVs in breast cancer diagnosis

*Alu* elements and LINE-1 are common retrotransposons in the human genome, serving a key role in genome stability, gene expression regulation and epigenetic modification. Their abnormal expression often serves as key molecular markers in the development and progression of tumors. In cfDNA, changes in *Alu* fragment copy number are closely associated with the occurrence and development of breast cancer. Furthermore, LINE-1 copy number ratio has been reported to differentiate patients with cancer from healthy individuals. Lee *et al* (132) analyzed plasma cfDNA from 26 patients with breast cancer and 10 healthy controls in 2019. The study reported that the area under the curve (AUC) value in diagnosing breast cancer by detecting low LINE-1 methylation levels was 0.78. Arko-Boham *et al* (133) further highlighted the diagnostic potential of *Alu* 115 and *Alu* 247 as biomarkers for breast cancer. DNA integrity was calculated by measuring the levels of these markers in the serum of 32 patients with breast cancer and 32 healthy controls. The study reported that levels of *Alu* 115 and *Alu* 247 were markedly elevated in patients with breast cancer, while DNA integrity was lower compared with that in controls. In 2023, Abd El Hafeez *et al* (134) conducted an experiment recruiting 20 healthy individuals, 20 patients with benign breast lesions and 60 patients with breast cancer. Results of the study indicated that the sensitivity of *Alu* 247 in serum for the diagnosis of breast cancer was 86.78%, the specificity was 75% and the AUC was 0.848. This study further confirmed the potential of *Alu* 247 and *Alu* 115 levels and DNA integrity in peripheral blood as markers for breast cancer screening and early diagnosis. In the same year, Bortul *et al* (135) used ddPCR technology to analyze plasma samples from 106 patients with breast cancer and 103 healthy women. The study evaluated the copy number ratio of *Alu* 260/111 bp and LINE-1 266/97 bp as well as cfDNA integrity. The results exhibited that the copy number ratios of *Alu* 260/111 and LINE-1 266/97 were markedly lower in the breast cancer group compared with that in the control group. Receiver operating characteristic analysis confirmed that these copy number ratios can effectively distinguish breast cancer cases from controls, with LINE-1 exhibiting notably increased diagnostic performance compared with *Alu*. Analysis of *Alu* and LINE-1 copy number ratios and DNA integrity in cfDNA provides a highly sensitive and specific method for distinguishing patients with breast cancer from healthy individuals. These markers demonstrated strong potential as non-invasive diagnostic tools. In addition, the methylation levels of *Alu* and LINE-1 further enhanced their application value in early breast cancer detection.

### Role of cfDNA gene mutations in the pathogenesis and early diagnosis of breast cancer

Breast cancer is a complex and heterogeneous disease whose development is driven by multiple genetic and environmental factors, including inherited mutations (e.g., BRCA1/BRCA2, TP53, PALB2), hormonal exposure (e.g., early menarche, hormone replacement therapy), reproductive history (e.g., nulliparity, late age at first pregnancy), lifestyle factors (e.g., high alcohol consumption, obesity) and ionizing radiation (136,137) Among them, 5–10% of breast cancer cases have a clear genetic predisposition. Germline or somatic mutations in specific genes serve a key role in the development and early screening of breast cancer. By analyzing the genomic sequences of cfDNA from patients with breast cancer, researchers can detect genetic abnormalities and use bioinformatics methods to assess the pathogenicity of these mutations. For example, a previous study confirmed that the V465M mutation of the SMAD4 gene is markedly associated with breast cancer. This mutation may enhance tumor invasion and metastasis by inhibiting the TGF-β signaling pathway (138). In addition, cfDNA-based ESR1 mutation detection has demonstrated high sensitivity and specificity, with an overall accuracy of 88.96%, a positive predictive value of 56.94% and a negative predictive value of 88.53%. This method is hypothesized to become a key diagnostic tool in identifying ESR1 mutations in patients with breast cancer (139). Recent studies have identified a variety of cfDNA gene mutations that can serve as potential biomarkers for breast cancer, which are expected to serve a key role in breast cancer screening and diagnosis in the future (140) [Table SI (141–149)]. The identification of these genes not only enhances current understanding of the molecular pathogenesis of breast cancer, but also provides novel avenues for early diagnosis and personalized treatment. Effective use of these genetic markers is hypothesized to notably improve the early detection rate and diagnostic accuracy of breast cancer, thereby providing patients with more timely intervention opportunities [Table SI (141–149)].

### Potential of breast milk in breast cancer screening in postpartum women

Due to the special physiological status of lactating women, there are certain limitations in the implementation of traditional biopsy methods. As an alternative, breast milk has emerged as a non-invasive liquid biopsy sample that exhibits notable potential for breast cancer screening in postpartum women. Saura *et al* (150) used ddPCR technology to analyze ctDNA in breast milk, finding that tumor mutations could be detected in 87% of cases, while in matched plasma samples, mutations failed to be detected in 92% of cases. This result suggested that ctDNA in breast milk is more sensitive compared with that in plasma in detecting breast cancer. In 2 patients, ctDNA was detected in breast milk 18 and 6 months before standard diagnosis, respectively, further highlighting its potential as an early screening and diagnostic tool for breast cancer (150). In addition, Cunningham and Turner (151) used NGS technology to simultaneously analyze 54 common breast cancer mutated genes in breast milk and blood samples. The results indicated that the sensitivity of ctDNA detection in breast milk was markedly higher compared with that in plasma. This confirmed the feasibility of breast milk ctDNA analysis and suggested that it may become an effective strategy for breast cancer screening in the future.

### Dynamic monitoring of efficacy and treatment guidance

The treatment strategy for breast cancer is mainly based on tumor stage, molecular characteristics and the overall physical condition of the patient. Tumor staging is a key factor in determining the direction of basic treatment, while molecular characteristics influence the selection of targeted therapy, endocrine therapy and chemotherapy regimens. Currently, standard treatments for breast cancer include surgical resection, radiotherapy, chemotherapy, targeted therapy and adjuvant therapy. In recent years, the development of targeted therapy has made precise intervention against cancer cell-specific molecular targets (mainly proteins) and driver gene mutations (such as PIK3CA, ESR1 and HER2) a reality (152), notably improving the prognosis of patients with breast cancer (153). However, effectively dealing with intra-tumor heterogeneity, dynamically changing therapeutic responses, and the generation and development of drug resistance is still a major clinical challenge (154). CDK4/6 inhibitors have become the standard treatment for ER^+^/HER2^−^ advanced breast cancer. In this context, monitoring ctDNA levels in patients with breast cancer aids in the real time assessment of treatment response. Continuous ctDNA analysis is a key tool in evaluating the efficacy of CDK4/6 inhibitors. For example, a previous study involving 33 patients with HR^+^/HER2^−^ metastatic breast cancer demonstrated that ctDNA analysis could detect disease progression months before radiographically visible changes, with a sensitivity and specificity of 75 and 92%, respectively (155). This finding suggested that continuous ctDNA monitoring can enable early detection of disease progression and real-time assessment of the efficacy of CDK4/6 inhibitors. To further analyze the genetic mechanisms associated with disease progression and drug resistance, ESR1 mutations detected in ctDNA have been identified as notable independent predictive markers of hormone therapy resistance. Notably, CDK4/6 inhibitors exhibit the ability to overcome ESR1-dependent drug resistance, which further highlights their clinical application value in the management of drug-resistant breast cancer (156).

### Predicting the efficacy of neoadjuvant chemotherapy (NAC)

Compared with other major breast cancer subtypes (HR^+^/HER2^−^, HER2^+^), TNBC is a highly aggressive and molecularly heterogeneous breast cancer subtype characterized by loss of ER, progesterone receptor and HER2 expression, resulting in relatively limited treatment options and poor prognosis. Due to the lack of endocrine or targeted therapy options, NAC has become the primary treatment for patients with TNBC. The treatment aims to shrink tumors before surgery, serving a key role in multidisciplinary breast cancer management. It has been reported that 40–50% of patients with TNBC achieve a pathological complete response (pCR) after NAC, which may enable breast-conserving surgery instead of mastectomy (157). In addition, multiple studies have confirmed that NAC can markedly improve the OS rate of patients with TNBC (158,159). Previous studies have highlighted the important clinical value of cfDNA in the context of NAC for TNBC. For example, Cirmena *et al* (160) analyzed plasma samples from 51 patients with breast cancer who received NAC in 2022 and evaluated the cfDNA integrity index (cfDI) by electrophoresis. The results demonstrated that cfDI has high sensitivity and specificity in predicting pCR. In addition, combining cfDI with MRI markedly improved the accuracy of pCR assessment. Another previous study reported that ctDNA clearance in the mid-term of NAC was notably associated with pCR, while persistent ctDNA positivity predicted poor OS and RFS. These results suggested that continuous ctDNA monitoring is considered to become a key biomarker for efficacy prediction and prognosis assessment in patients with TNBC (161). A previous study by Parsons *et al* (162) in 2024 further confirmed that after NAC, the median ctDNA tumor score of patients with TNBC who responded to treatment exhibited a 285-fold reduction, while that of non-responders exhibited only a 24-fold reduction. Furthermore, 58% of patients had ctDNA levels that reduced below commercial detection thresholds, which strongly supports the potential of ctDNA as a valid indicator in assessing treatment response. Furthermore, previous studies confirmed that ctDNA detection after NAC was associated with a higher risk of recurrence, revealing a notable difference in event-free survival. During follow-up, ctDNA was not detected in non-relapsed cases, but was more commonly detected in relapsed patients. In addition, ctDNA detection can precede clinical recurrence by up to 13 months, highlighting its clinical feasibility in monitoring early breast cancer recurrence after NAC (163,164). In summary, ctDNA has exhibited notable clinical value in the efficacy evaluation, recurrence risk prediction and long-term prognosis judgment of NAC for TNBC, providing a key molecular basis for clinical decision-making.

### Early warning role in breast cancer disease progression and recurrence monitoring

CfDNA detection provides a highly sensitive and specific non-invasive method for disease monitoring in patients with breast cancer. Notably, ctDNA can be detected up to 2 years before the onset of distant metastatic recurrence, with notably increased predictive power compared with traditional imaging examinations, CA15-3 biomarker analysis, clinical examinations and liver function tests. Furthermore, elevated ctDNA levels are associated with poor prognosis and detection of ctDNA mutations before treatment often predicts unfavorable survival outcomes (165,166). In the majority of patients with breast cancer, ctDNA can be detected months before clinical recurrence with extremely high specificity. This advance detection provides a key time window for the timely introduction of non-cross-resistant therapies, thereby effectively preventing or delaying clinically visible metastasis and recurrence. Further research confirmed that ctDNA analysis can effectively predict the recurrence of all major breast cancer subtypes, with a detection time on average 10.7 months earlier than clinical recurrence (167). Furthermore, the failure to clear ctDNA after treatment is a key predictor of adverse outcomes, including distant metastasis and local recurrence. Notably, in patients who did not achieve pCR, a higher rate of ctDNA clearance was markedly associated with improved survival (168). Continuous monitoring of ctDNA enables the dynamic, real-time assessment of treatment response, providing unique clinical opportunities for treatment adjustments aimed at delaying metastatic recurrence and improving patient outcomes. In brain metastatic breast cancer, ctDNA can be detected in both cerebrospinal fluid and plasma. A previous study involving 30 patients with breast cancer with leptomeningeal metastases employed ultra-low-depth WGS technology to evaluate ctDNA scores. The results demonstrated that ctDNA was present in the cerebrospinal fluid of all patients with brain metastases, regardless of negative cytology results or borderline MRI findings. During intrathecal therapy, continuous ctDNA monitoring revealed that declining ctDNA levels were markedly associated with prolonged survival, while elevated ctDNA could be detected up to 12 weeks before clinical progression (169). Overall, ctDNA, as a non-invasive biomarker, exhibits strong potential in quantitatively monitoring treatment response and disease progression in brain metastatic breast cancer, aiding in the optimization of early intervention strategies and guide personalized precision treatment.

### Role of genetic testing in the evaluation and monitoring of breast cancer efficacy

In breast cancer response evaluation and disease monitoring, cfDNA-based analysis of specific gene mutations is increasingly becoming a key biomarker. Specific genetic mutations in cfDNA not only provide a notable basis for personalized treatment plans, but also enable dynamic, real-time tracking of disease progression and treatment response, therefore optimizing clinical decisions and improving patient outcomes. A previous study involving 255 patients with stage IV breast cancer reported that 89% had at least one genetic mutation detected in their ctDNA samples. Specifically, in HR^+^ patients, the most common mutated genes are PIK3CA, ESR1 and TP53; in HER2^+^ patients, TP53, PIK3CA and Erb-b2 receptor tyrosine kinase 2 (ERBB2) mutations are the most common, with ERBB2 changes mainly manifested as CNVs; in patients with TNBC, TP53 and PIK3CA mutations dominate, and CNVs of Myc, cyclin E1 and PIK3CA are often observed. Across the entire patient cohort, the mutation rates of PIK3CA, ESR1 and ERBB2 were 39.6, 16.5 and 21.6%, respectively (170). Recent studies have further emphasized the key role of cfDNA genetic alterations in breast cancer treatment monitoring, with PIK3CA and ESR1 being the most commonly used molecular markers in assessing disease progression and treatment response (171). Table SII (172–182) summarizes the applications of these cfDNA-based genetic tests in breast cancer management, demonstrating their growing clinical value.

The studies mentioned in Table SII demonstrated that the gene mutation spectrum detected in blood and tissue samples was highly consistent. The VAF and the number of mutations were markedly associated with the number of metastatic sites. These findings not only highlighted the genetic heterogeneity of metastatic breast cancer but also highlighted the high detection rate of clinically actionable mutations in blood samples (170). As a non-invasive measure of tumor burden, the quantitative detection of genetic mutations in breast cancer stem cells using cfDNA provides a reliable molecular basis in assessing disease progression (183). In patients with breast cancer, PIK3CA hotspot clonal missense mutations and cyclin D1 gene amplification are commonly identified in primary tumors. Notably, following treatment with alpelisib combined with letrozole, these mutations became undetectable in cfDNA, indicating a favorable response to treatment (184). Similarly, another study used NGS to analyze primary breast tumors and identified multiple mutations, including PIK3CA alterations. After 6 years of treatment with trastuzumab and nab-paclitaxel, cfDNA analysis did not detect mutations associated with the primary tumor, suggesting that the patient may have achieved molecular remission (185). Collectively, these findings highlighted the key clinical utility of cfDNA monitoring in assessing treatment efficacy and disease progression. Breast cancer recurrence remains a major clinical challenge, with ~30% of patients experiencing recurrence after initial treatment. Monitoring treatment response is particularly key in pregnant patients, as traditional approaches may have limitations. A previous study compared the effectiveness of whole-genome NIPT technology in detecting cancer-specific CNVs in ctDNA in 25 pregnant and non-pregnant women. The results revealed that pregnant patients had higher sensitivity of ctDNA, confirming the clinical feasibility of ctDNA as a non-invasive tool in monitoring treatment response in this specific patient population (186).

### Prognostic assessment and clinical risk stratification of breast cancer

The prognosis assessment of breast cancer mainly relies on clinicopathological characteristics and molecular markers. However, accurate prognostic assessment still faces several challenges due to the complexity of tumor heterogeneity and difficulty in detecting early recurrence. Although the detection rate of early-stage breast cancer is high, ~20% of patients still experience recurrence after receiving standard treatment (187). Therefore, accurate assessment of patient prognosis is key to optimizing subsequent management and improving patient outcomes. In operable breast cancer, univariate analysis results revealed that the detection of ctDNA at baseline, after neoadjuvant therapy and during follow-up was markedly associated with worse disease-free survival (DFS) and OS (188). In addition, a high baseline ctDNA CNVs burden was confirmed to be associated with worse OS and RFS (189). Sustained increases in ctDNA levels after treatment strongly predict worse OS (190). In a cohort of patients with primary breast cancer who underwent surgery and adjuvant chemotherapy, ctDNA^+^ patients had notably shorter progression-free survival (PFS) compared with ctDNA^−^ patients. Furthermore, mutational heterogeneity of ctDNA was negatively associated with PFS. In TNBC, ctDNA was detected positive in all relapsed patients within a median of 8 months, whereas non-relapsed patients remained ctDNA negative during a median follow-up of 58 months. ctDNA positivity has been reported to be highly associated with shorter RFS and OS (191,192). A previous study involving 84 patients with high-risk early-stage breast cancer treated with NAC or NAC combined with MK-2206 (an AKT inhibitor) demonstrated that the ctDNA positivity rate was highest before treatment and gradually decreased as the treatment progressed. More notably, ctDNA clearance after treatment was closely associated with pCR and the prognosis of ctDNA^−^ patients who did not reach pCR was markedly improved compared with that of ctDNA^+^ patients (168). Persistent ctDNA positivity is considered a notable predictor of adverse outcomes and metastatic recurrence, further confirming the key clinical value of ctDNA detection in guiding treatment decisions.

### Dynamic monitoring value of ctDNA in predicting breast cancer recurrence and survival

Previous studies have reported that ctDNA testing can detect recurrence earlier than imaging and clinical symptoms, with an average lead time of 3.81 months. In patients with primary breast cancer who receive postoperative adjuvant chemotherapy, ctDNA is often detected before clinical or imaging confirmation of recurrence, with a maximum predictive time window of up to 38 months (152,193). Serial postoperative ctDNA assessment not only provides valuable prognostic information but also creates a valuable time window for early therapeutic intervention. The sensitivity range of ctDNA in detecting breast cancer recurrence ranges from 0.31 to 1.0 and its specificity ranges from 0.7 to 1.0. On average, it can confirm recurrence 10.81 months earlier than imaging (188). Changes in ctDNA levels can indicate disease progression weeks before imaging findings, making it a notable biomarker for real-time monitoring of disease status. In metastatic breast cancer, ctDNA levels dynamically reflect tumor burden and progression prior to treatment. The study has reported that a high tumor burden index before treatment was markedly associated with shorter OS, while a reduction to <0.02% during treatment was associated with longer PFS and OS (194). In addition, the tumor burden index can effectively distinguish treatment response (complete response/partial response) from disease progression. Notably, ctDNA tumor fraction ≥10% in metastatic breast cancer has been reported to be markedly associated with OS and can serve as an independent prognostic factor in multivariate analysis (195). Chromosomal instability (CIN) is a hallmark feature of tumor development and progression. By driving genomic instability, it endows tumor cells with a growth advantage, enhances anti-apoptotic ability and promotes the development of drug resistance. CIN can be quantified by somatic CNVs, MSI and other chromosomal structural changes. In patients with breast cancer, cfDNA CIN is associated with prognosis. Related studies have reported that the median OS and PFS of patients with metastatic breast cancer with high cfDNA CIN are markedly lower compared with those with low CIN score (196). In addition, cfDNA CIN in patients with recurrent breast cancer demonstrated high accuracy in recurrence monitoring, with a positive rate of 77.6%, outperforming traditional biomarkers CA15-3 and CEA. When combined with traditional markers, the positive rate can be further increased to 88.7%. Particularly in patients with shorter DFS, cfDNA CIN exhibits high prognostic predictive value (197). Further analysis of cfDNA in patients with early-stage breast cancer reported that a high cfDNA mutation burden was associated with worse RFS. Despite the inconsistency between tumor tissue and cfDNA samples, specific somatic variants in cfDNA are still associated with worse RFS, suggesting that cfDNA mutation burden can serve as a prognostic marker in patients with early-stage breast cancer (198). Among patients with metastatic TNBC, cfDNA tumor fraction is detectable in 96.3% of cases, with 63.9% exhibiting somatic CNVs. Notably, cfDNA tumor fraction ≥10% is markedly associated with worse metastasis-free survival. Furthermore, specific somatic CNVs are highly expressed in patients with metastatic TNBC and exhibit strong prognostic predictive value (199). Overall, the detection of high allele frequencies and a large number of non-synonymous mutations in cfDNA of patients with metastatic breast cancer has been confirmed to be markedly associated with poor OS (200). As key liquid biopsy biomarkers, cfDNA and ctDNA serve a differentiated role in predicting treatment response and evaluating prognosis of different breast cancer subtypes. Analyzing its manifestations can provide notable insights into breast cancer management. In patients with TNBC, cfDNA concentration measured 3 weeks before treatment was inversely associated with residual cancer burden (RCB), whereas ctDNA concentration was positively associated with RCB at all time points examined. In patients with HR^+^/HER2^−^ breast cancer, cfDNA concentration was not associated with NAC response; however, patients with high cfDNA concentrations had lower distant RFS compared with those with low concentrations. By contrast, in patients with TNBC, cfDNA concentration had no notable effect on survival, whereas ctDNA was associated with RFS at all time points (201). In summary, compared with cfDNA, ctDNA concentration is considered a more sensitive and clinically practical prognostic predictor, serving as a key tool in monitoring treatment response and guiding clinical decision-making.

### Prognostic value of circulating tumor cells (CTCs) and cfDNA

CTCs are cancer cells that detach from primary tumors or metastatic sites and enter the blood circulation, reflecting the invasion and metastasis potential of tumors. The combined application of CTCs, cfDNA and ctDNA can improve the accuracy of breast cancer prognosis assessment and provide a key basis in formulating personalized treatment plans. Previous studies have reported that both CTCs and ctDNA are key prognostic markers. There were notable differences in PFS and OS based on CTC count and ctDNA levels. Patients with CTC ≥5 or ctDNA percentage ≥0.5, as well as those carrying ≥2 mutations, have markedly worse clinical outcomes compared with patients below these thresholds (202). Furthermore, both total cfDNA content and CTC count can serve as predictors of OS, while total cfDNA levels alone can predict PFS and disease response. Notably, the combined analysis of CTCs and cfDNA has been reported to provide more accurate information for prognostic assessment compared with traditional biomarkers such as CA15-3 and alkaline phosphatase (203). Several studies have further demonstrated that MET upregulation in metastatic sites is markedly higher compared with that in primary tumors and MET^+^ CTCs, elevated cfDNA levels and ESR1 mutations have all been reported to be closely associated with poor prognosis (204–206). The integrated analysis confirmed that CTC count ≥5 and high cfDNA levels are markedly associated with shortened PFS and OS. When high CTC and high cfDNA levels coexist, the risk of death for the patient is notably increased (207). Furthermore, longitudinal studies have reported that cfDNA levels can explain differences in prognostic assessment based on CTCs. The 24-month DFS probability was 52% for patients positive for both ctDNA and CTCs, compared with 89% for patients negative for both ctDNA and CTCs. The mere presence of ctDNA positivity is also associated with a lower probability of DFS compared with ctDNA^−^ cases (208). The combined detection of ctDNA and CTCs improves the sensitivity and accuracy of breast cancer prognosis assessment and provides key clinical insights for early intervention and personalized treatment strategies in the future.

### In-depth analysis of the relationship between gene mutations and prognosis

Advances in early cancer detection technology and the continued development of targeted anti-breast cancer therapies have markedly increased the 5-year survival rate of breast cancer to ~90% (209). However, despite achieving complete remission after initial treatment, ~30% of patients will eventually experience disease recurrence. Furthermore, the 5-year survival rate for advanced breast cancer is still low, at ~30% (210,211). This high recurrence rate and low survival rate is largely attributed to the complex genetic background of breast cancer, which continues to pose notable challenges for its complete eradication. Current research focuses on assessing patient survival outcomes by detecting specific genomic alterations in cfDNA. These studies typically involve the extraction of genomic DNA from tumor tissue or liquid biopsy samples to identify genetic biomarkers with prognostic predictive value. In addition to traditional clinical and histological factors, these genetic markers are increasingly being considered as notable adjunct tools for prognostic assessment (212–214). To gain further understanding of the relationship between gene mutations and breast cancer prognosis, the present review summarized recent studies on cfDNA and ctDNA mutations and their impact on survival outcomes [Table SIII (215–225)].

The present section focused on elucidating the detection results and clinical relevance of different gene mutations in patients with breast cancer. These findings provide novel perspectives and strong evidence for personalized treatment strategies and improved prognostic assessment. From the aforementioned studies, it is evident that the detection of genetic mutations and their relationship with patient prognosis have become a core area of breast cancer research. Different gene mutations can affect the occurrence, development and response to treatment in breast cancer, thereby affecting the survival outcome of the patient. Table SIV (215–225) summarizes the key genes and their functions that have been explored in multiple studies investigating the relationship between gene mutations and various survival indicators. The present review observed that gene mutations such as TP53, ESR1 and PIK3CA have been confirmed to be markedly associated with poor breast cancer survival outcomes in multiple studies. In addition, certain less studied but equally key genes [such as plasmacytoma variant translocation 1 (PVT1), CCCTC-binding factor (CTCF), guanine nucleotide binding protein, α stimulating activity polypeptide (GNAS) and Notch1] have also been identified to be closely associated with the prognosis of patients with breast cancer (226–229).

## 6. Translation dilemma and future strategies for cfDNA in the precise diagnosis and treatment of breast cancer

cfDNA, as a non-invasive liquid biopsy technology, has demonstrated notable potential in the field of precise diagnosis and treatment for breast cancer. Currently, the field is at a key juncture transitioning from ‘technical verification’ to ‘clinical integration’ (115,213) The core focus lies in constructing an integrated evidence chain that encompasses multi-dimensional analysis of cfDNA, standardized processes and clinical utility verification.

### Multidimensional challenges and integration dilemmas in clinical translation of cfDNA

CfDNA faces multiple challenges at the biological, technical and clinical application levels during the clinical translation of breast cancer. These challenges are intertwined and together constitute a complex dilemma for its integration into clinical practice.

### Fundamental contradiction between biological characteristics and detection sensitivity

The presence of cfDNA in blood is a complex biological process. In early-stage breast cancer, tumor-derived ctDNA typically constitutes <0.01% of the total cfDNA, posing a fundamental challenge in the detection of extremely low-abundance signals (19,230). The release mechanism of cfDNA is complex and diverse, including not only apoptosis and necrosis of tumor cells, but also various factors such as active cell secretion, dynamic changes in the tumor microenvironment and the systemic physiological state of the host (such as inflammatory response and strenuous exercise). These non-tumor-derived cfDNA collectively constitute notable biological background noise, markedly increasing the difficulty of tumor-specific cfDNA detection (231). In addition, the spatiotemporal heterogeneity of tumors implies that a single liquid biopsy may not fully capture the characteristics of all subclones. It may particularly miss tumor cells with weak hematogenous metastasis capabilities or those located in specific microenvironments (such as brain metastases), potentially resulting in differences with tissue biopsy results (198). Therefore, it is necessary to carefully evaluate the precise positioning of cfDNA analysis in specific clinical scenarios: Whether it serves as an independent alternative diagnostic tool or as an effective complementary monitoring method for existing methods warrants further research.

### Difficulties in selecting technology platforms and lack of standardization

cfDNA analysis technology faces a dilemma of multiple choices. dPCR has high sensitivity in detecting known low-frequency variants but lacks the ability to explore unknown variants (232,233). NGS enables broad-spectrum screening, yet it still has limitations in ultra-low abundance detection, cost control and complexity of bioinformatics analysis (234). However, a more fundamental and urgent challenge lies in the lack of a standardized system for the entire cfDNA detection workflow. From sample collection (such as anticoagulant selection), pretreatment (such as centrifugation parameters), cfDNA extraction, library construction and downstream bioinformatics analysis, there is a lack of unified and extensively accepted standard operating procedures across all key stages of the chain. This lack of standardization restricts the reproducibility and comparability of different study results and thus, reduces the clinical reliability of cfDNA detection (235). Therefore, establishing and implementing an internationally recognized ‘clinical-grade’ cfDNA testing quality management system is the cornerstone and prerequisite for its successful translation from technology to clinical practice.

### Complexity of data interpretation and clinical integration

**Figure 4.**
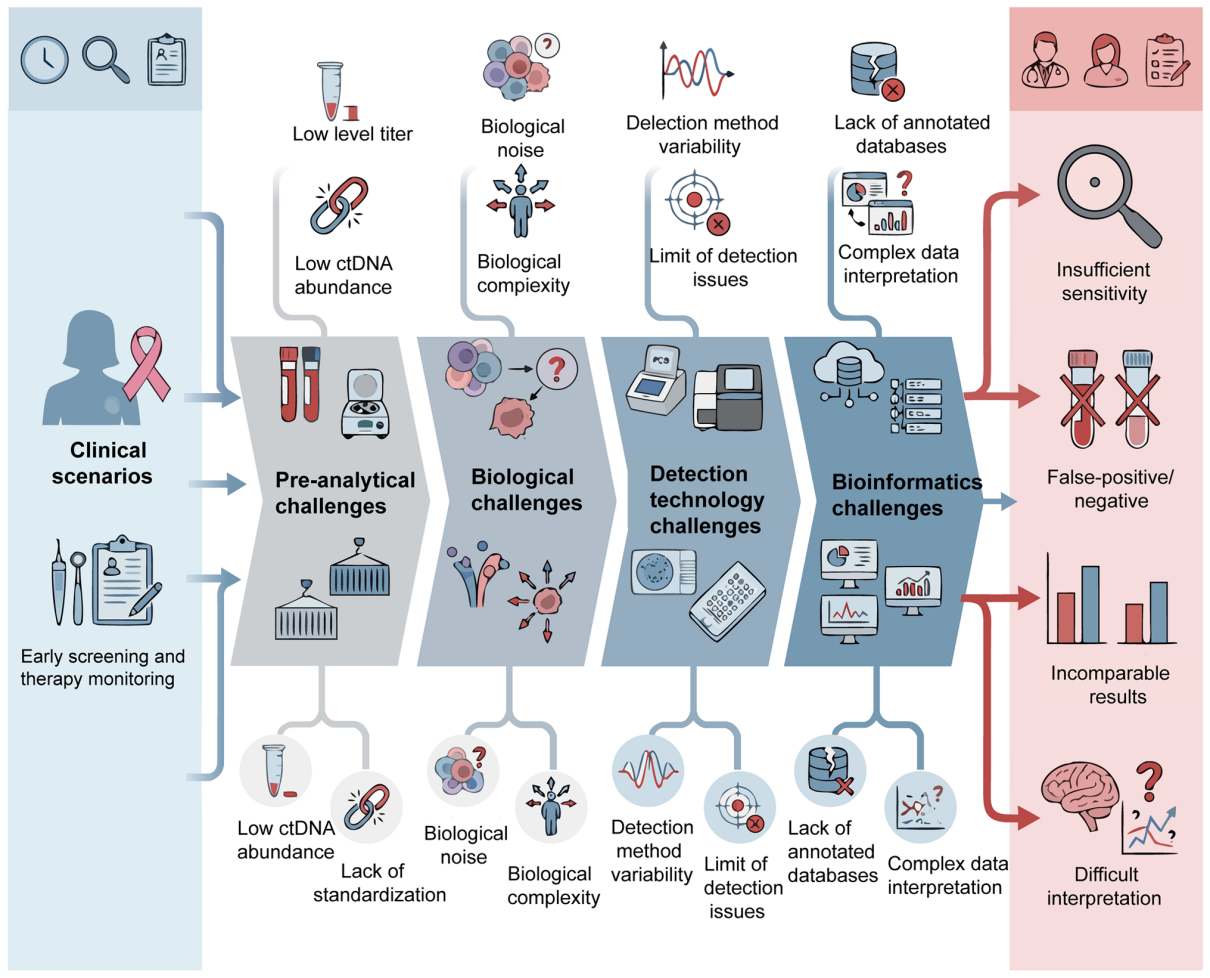
Visual representation of the main challenges encountered in each association and their interrelationships in the entire chain from circulating cell-free DNA sample collection to clinical decision-making.

Obtaining the cfDNA variation profile is the starting point for analysis. The accurate interpretation of its further clinical significance faces more complex and formidable challenges. The core challenge lies in accurately distinguishing background mutations from non-tumor sources such as tumor driver mutations, non-functional passenger mutations and CH (CH of indeterminate potential). This relies heavily on continuously updated and improved clinical annotation databases (236). A more fundamental challenge is how to effectively transform complex and dynamically changing cfDNA data into specific, actionable clinical action guidelines. For example, the scientific definition of a ‘positive’ threshold for molecular residual disease (MRD) testing remains a key issue that needs to be resolved. In addition, whether early or preemptive intervention strategies based on dynamic changes in cfDNA can markedly improve the long-term survival outcomes of patients still needs to be verified by high-level evidence-based medical evidence. However, there is still a lack of prospective interventional clinical studies that can provide high-level evidence-based evidence (237). In order to achieve the deep integration of cfDNA data with clinical decision-making pathways, it is key to establish a multidisciplinary and cross-institutional collaboration mechanism to jointly formulate rigorous and evidence-based clinical practice guidelines b. In order to systematically explain the association and logic between the aforementioned multi-dimensional challenges, the present review presents a schematic diagram of the integration dilemma of cfDNA clinical translation (Fig. 4).

### Staged path for future development

To systematically promote the application of cfDNA in breast cancer management, the present review proposed a staged transformation strategy guided by the disease progression stage of breast cancer and the maturity of cfDNA technology.

### A near-term feasible direction: Clinical verification in the management of advanced breast cancer

The short-term goal is to establish the clinical utility of cfDNA analysis as a routine and reliable means of disease monitoring in patients with metastatic breast cancer as a priority. The core theoretical basis of this strategy is that patients with metastatic breast cancer usually have a higher tumor burden, making the cfDNA detection signal more notable. Furthermore, there is an urgent and clear need for non-invasive, real-time, dynamic monitoring methods in clinical practice. The key transformation path at this stage focuses on the following three core aspects: First, accurately verify the leadership and accuracy of cfDNA dynamic changes in predicting imaging response and disease progression through prospective studies; second, build a systematic monitoring system to track the evolution of acquired mutations in key driver genes (such as ESR1 and PIK3CA) in real time during treatment; lastly, develop an accurate prognostic stratification model based on cfDNA characteristics and optimize the screening strategy for patients in clinical trials. The milestone in achieving this stage of transformation will be the successful completion of at least one prospective randomized controlled clinical trial. This trial needs to demonstrate that treatment regimen adjustments based on dynamic changes in cfDNA can markedly and statistically improve the long-term survival outcomes of patients.

### Mid-term translation direction: Decision support for adjuvant treatment of early breast cancer

Currently, with the sensitivity of detection technology reaching ≤0.001% levels, the technical feasibility of cfDNA analysis in MRD detection has been initially and encouragingly verified. The core task of this stage is to further integrate cfDNA detection technology into the clinical decision-making process for adjuvant treatment and neoadjuvant treatment in early-stage breast cancer. Its key tasks include: First, verifying the validity and reliability of cfDNA clearance status after neoadjuvant therapy as a surrogate endpoint for pCR; second, establishing a standardized operating plan for postoperative MRD monitoring and scientifically defining its threshold and timing for clinical intervention. The key sign in achieving transformation at this stage is confirmation, through multiple large-scale prospective cohort studies, that adjuvant treatment de-escalation or escalation strategies guided by ctDNA MRD can optimize patient prognosis and reduce unnecessary treatment toxicity.

### Long-term exploration direction: Precise screening system for high-risk groups

The long-term goal is to use cfDNA technology to build an accurate, non-invasive and efficient early screening system for high-risk groups of breast cancer. This requires overcoming the current challenge of insufficient sensitivity of cfDNA in early-stage tumor detection and integrating it with deep multi-omics analysis (such as cfDNA methylation, fragmentation characteristics and proteomics). The main tasks of this stage include: First, developing ultra-high-sensitivity multi-omics cfDNA detection technology to capture weak tumor signals at an early stage; second, establishing a large-scale prospective cohort study to verify the specificity and sensitivity of cfDNA in early breast cancer screening in asymptomatic high-risk groups; lastly, systematically evaluating ethical issues, social acceptance and economic benefits to ensure the sustainability and fairness of the screening system. The ultimate goal of achieving this stage of transformation is for cfDNA to potentially become a routine component of early breast cancer screening, markedly improve the early diagnosis rate and thereby reduce breast cancer mortality in the future.

### Future prospects and challenges

cfDNA has broad application prospects in the field of precision diagnosis and treatment of breast cancer; however, it requires continuous investment and interdisciplinary collaboration in the future.

### Multi-omics integration and artificial intelligence assistance

The future development trend will involve the multi-omics integration of cfDNA with other liquid biopsy markers (such as CTCs, exosomes and circulating RNA) as well as traditional imaging and pathology data. Combining artificial intelligence and machine learning algorithms, it is expected to extract further biological information from massive complex data, establishing more accurate prediction models and decision support systems. For example, the application of deep learning to analyze cfDNA fragmentation patterns and methylation profiles may further improve the accuracy of early diagnosis and MRD detection.

### Ethical, social and economic impacts

As cfDNA becomes more extensively applied, the possible ethical, social and economic impacts need to be addressed. For example, issues such as over-diagnosis, patient anxiety, data privacy protection, testing costs and allocation of medical resources all require further discussion and regulation alongside technological development. Establishing a sound regulatory framework and ethical review mechanism to ensure the responsible application of technology is key to the sustainable development of cfDNA.

### International cooperation and data sharing

Promoting the global clinical transformation of cfDNA technology requires strengthening international cooperation and data sharing. Establishing a unified biobank, shared clinical data and bioinformatics analysis platform will accelerate the identification and validation of novel biomarkers, as well as the development of clinical practice guidelines in the future.

## 7. Conclusion

CfDNA technology has exhibited notable potential in the precise diagnosis and treatment of breast cancer; however, its clinical translation may not be immediate. By confronting the multi-dimensional challenges at the biological, technical and clinical integration levels, and adopting a staged translation strategy, focusing on the monitoring of advanced breast cancer, decision support for adjuvant treatment of mid- and early-stage breast cancer and thus, exploring the precise screening of high-risk groups, can progressively transition cfDNA from the laboratory to the clinic. In the future, with the deepening of multi-omics integration, artificial intelligence assistance and international cooperation, cfDNA is hypothesized to become a key tool in breast cancer management. This progress potentially offers patients more precise and personalized treatment plans and therefore, improves the survival and quality of life for patients with breast cancer worldwide.

## Supplementary Material

Supporting Data

## Data Availability

Not applicable.

## Abbreviations

cfDNA: circulating cell-free DNA
ctDNA: circulating tumor DNA
CTCs: circulating tumor cells
cf-mtDNA: circulating mitochondrial DNA
cf-fDNA: cell-free fetal DNA
ddPCR: droplet digital PCR
NGS: next-generation sequencing
ER: estrogen receptor
cfDI: cfDNA integrity index
OS: overall survival
PFS: progression-free survival
DFS: disease-free survival
RFS: recurrence-free survival
MRD: molecular residual disease
TNBC: triple-negative breast cancer
CNVs: copy number variations
MSI: microsatellite instability
VAF: variant allele frequency
CH: clonal hematopoiesis
CIN: chromosomal instability
pCR: pathological complete response
NAC: neoadjuvant chemotherapy
NIPT: non-invasive prenatal testing
RCB: residual cancer burden
