# Supplementary material for: Biological basis and clinical translation prospects of circulating cell-free DNA in precision management of breast cancer (Review)
Source: Oncol Lett. 2026 Apr 2;31(6):215. doi: 10.3892/ol.2026.15570 (PMC13088249; doi:10.3892/ol.2026.15570)
Supplement: Supporting Data [file Supplementary_Data.pdf]

## Supplementary methods

### Data S1. Literature search strategy and screening process.

In order to comprehensively and systematically evaluate the biological basis and clinical translation prospects of circulating cell-free DNA (cfDNA) in the precision management of breast cancer, the present study adopted a narrative review method. The core evidence comes from the systematic search and screening of the PubMed database, aiming to build a transparent, repeatable and cutting-edge evidence chain to provide solid support for the writing of each core chapter of the review.

*Search the database*. Main database: PubMed (https://pubmed.ncbi.nlm.nih.gov/).

*Search strategy construction*. The search will be conducted intensively in September 2024. The search strategy was designed around the core framework of the present review, namely biological properties-detection technology-clinical translation application, using a comprehensive search strategy from face to point. Core search logic: [cfDNA/circulating tumor DNA (ctDNA)] AND (biology) AND (breast cancer) AND (technology) AND (clinical application).

*Specific search terms and keyword combinations*. In order to take into account both the recall rate and the precision rate, the Medical Subject Headings (MeSH Terms) and free words (Title/Abstract Words) of the U.S. National Library of Medicine were comprehensively used, and combined through Boolean logical operators (AND, OR, NOT). During the search process, the present review divided it into four core concept modules A, B, C and D. Each module contains MeSH subject headings and free words.

*Circulating cell-free DNA*. (Circulating cell-free DNA[MeSH Terms]) OR (Cell-Free DNA[Title/Abstract])) OR (Cell Free DNA[Title/Abstract])) OR (DNA, Cell-Free[Title/Abstract])) OR (cfDNA[Title/Abstract])) OR (Circulating cell-free DNA[Title/Abstract]).

*Biological characteristics*. (biological phenomena[MeSH Terms] OR phenomena biological[Title/Abstract] OR biologic phenomena[Title/Abstract] OR (Phenomena[Title/Abstract] AND Biologic[Title/Abstract]) OR biological phenomenon[Title/Abstract] OR phenomenon biological[Title/Abstract] OR biological processes[Title/Abstract] OR processes biological[Title/Abstract] OR biological process[Title/Abstract] OR process biological[Title/Abstract]).

*Technology*. ((Detection technology[MeSH Terms] AND (y_10[Filter])) OR ((((High-Throughput Nucleotide Sequencing[MeSH Terms]) OR (Next-Generation Sequencing[Title/Abstract])) OR (Next Generation Sequencing[Title/Abstract])) OR (Sequencing, Next-Generation[Title/Abstract])) OR (High-Throughput DNA Sequencing[Title/Abstract]) AND (y_10[Filter]))) OR ((Polymerase Chain Reaction[MeSH Terms]) AND (droplet digital PCR[Title/Abstract] OR droplet digital PCR (ddPCR)[Title/Abstract] OR digital PCR[Title/Abstract]).

*Breast cancer*. ((((‘Breast Neoplasms’[Mesh]) OR ((Breast Neoplasm[Title/Abstract])OR(Neoplasm, Breast[Title/Abstract])OR(Neoplasms, Breast[Title/Abstract])OR(Breast Tumors[Title/Abstract])OR(Breast Tumor[Title/Abstract])OR(Tumor, Breast[Title/Abstract])OR(Tumors, Breast[Title/Abstract])OR(Breast Cancer[Title/Abstract])OR(Cancer, Breast[Title/Abstract])OR(Cancer of Breast[Title/Abstract])OR(Cancer of the Breast[Title/Abstract])OR(Malignant Neoplasm of Breast[Title/Abstract])OR(Breast Malignant Neoplasm[Title/Abstract])OR(Breast Malignant Neoplasms[Title/Abstract])OR(Malignant Tumor of Breast[Title/Abstract])OR(Breast Malignant Tumor[Title/Abstract])OR(Breast Malignant Tumors[Title/Abstract])OR(Mammary Cancer[Title/Abstract])OR(Cancer, Mammary[Title/Abstract])OR(Cancers, Mammary[Title/Abstract])OR(Mammary Cancers[Title/Abstract])OR(Mammary Neoplasms, Human[Title/Abstract])OR(Human Mammary Neoplasm[Title/Abstract])OR(Human Mammary Neoplasms[Title/Abstract])OR(Neoplasm, Human Mammary[Title/Abstract])OR(Neoplasms, Human Mammary[Title/Abstract])OR(Mammary Neoplasm, Human[Title/Abstract])OR(Breast Carcinoma[Title/Abstract])OR(Breast Carcinomas[Title/Abstract])OR(Carcinoma, Breast[Title/Abstract])OR(Carcinomas, Breast[Title/Abstract])OR(Mammary Carcinoma, Human[Title/Abstract])OR(Carcinoma, Human Mammary[Title/Abstract])OR(Carcinomas, Human Mammary[Title/Abstract])OR(Human Mammary Carcinomas[Title/Abstract])OR(Mammary Carcinomas, Human[Title/Abstract])OR(Human Mammary Carcinoma[Title/Abstract]))).

i) Diagnosis/Screening Module. (((((Mass Screening[MeSH Terms])) OR (Mass Screenings[Title/Abstract])) OR (Screening[Title/Abstract])) OR (Screenings[Title/Abstract])) OR ((((Early Diagnosis[MeSH Terms]) OR (Diagnosis, Early[Title/Abstract])) OR (Early Detection of Disease[Title/Abstract])) OR (Disease Early Detection[Title/Abstract]).

ii) Efficacy monitoring. ((((((Treatment Outcome[MeSH Terms]) OR (Disease Management[MeSH Terms])) OR (Precision Medicine[MeSH Terms])) OR (response monitoring[Title/Abstract])) OR (adaptive therapy[Title/Abstract])) OR (guide therapy[Title/Abstract])) OR (treatment guidance[Title/Abstract]).

iii) Prognosis. (((((Prognosis[MeSH Terms]) OR (Prognoses[Title/Abstract])) OR (Prognostic Factors[Title/Abstract])) OR (Prognostic Factor[Title/Abstract])) OR (Factor, Prognostic[Title/Abstract])) OR (Factors, Prognostic[Title/Abstract]).

*Search time range and filtering criteria*. Time range: January 1, 2016 to December 31, 2025. The present review chose 2016 as the starting point because since then, the application of ddPCR and next generation sequencing (NGS) technology in cfDNA analysis has begun to mature and a large number of related high-quality clinical studies have emerged. The year 2025 is included to incorporate Early View articles that have been published online. Inclusion criteria were as follows: i) Original research articles focusing on human breast cancer (including retrospective, prospective cohort studies and clinical trials); ii) high-quality systematic reviews or meta-analyses (for background introduction and argument

support); iii) the content directly involves the biology and detection technology of cfDNA/ctDNA or its application in breast cancer diagnosis, treatment monitoring and prognosis assessment; and iv) published in English. Exclusion criteria were as follows: i) Case reports, conference abstracts, editorials, reviews; ii) non-English literature; iii) the research focus is only on other biomarkers (such as circulating tumor cells, exosomes) and does not involve cfDNA; and iv) non-breast cancer research or only *in vitro* or animal experiments without involving patient samples.

*Literature screening and management process*

*Preliminary deduplication and merging.* After searching in each of the four modules namely, A, B, C and D, each module will randomly export 200 documents after searching and then import the results of each search query into the EndNote document management software to automatically remove duplicate records.

*Two-person independent screening.* In the first stage, two reviewers independently reviewed the titles and abstracts of all records based on the inclusion/exclusion criteria. Any vote passed will advance to the next stage. In the second stage, the full texts of the documents that passed the first stage were obtained and two reviewers independently conducted detailed evaluations to determine the final included documents.

*Disagreement resolution.* When disagreements arise at any stage, the two reviewers will resolve them through discussion and negotiation. If consensus still cannot be reached, the decision will be made by arbitration by a third senior researcher.

*Information extraction and evidence integration.* From the final included literature, systematically extract the following information: research design, sample type, detection technology, key gene mutations (such as ESR1, PIK3CA and TP53), clinical endpoints (for example, pCR, PFS and OS) and main conclusions. This information was classified, integrated and directly used to support the discussion of the main body of the review and formed Table SI (genes associated with pathogenicity/early diagnosis), Table SII (genes associated with efficacy monitoring), Tables SIII and IV (genes associated with prognosis and survival outcomes) in the article.

*Targetedness of search strategy*

*Chapter 1 and 2: Biology and subtypes.* Through the A and B search modules, research on biological characteristics such as cfDNA release, fragmentation and methylation, as well as literature on ctDNA, cf-mitocondrial DNA, cf-fetal DNA and other subtypes can be extensively obtained.

*Chapter 3: Detection technology.* Through the concept set A and C search modules, focus on the technical principles, comparative advantages, standardization challenges and emerging technologies of ddPCR and NGS.

*Chapter 4: Clinical translation.* Through each subset of the A and D search modules, the evidence supporting 'early screening', 'efficacy monitoring (including neoadjuvant therapy)', 'prognosis assessment and relapse warning' and 'resistance mechanism' are respectively searched to provide direct references for the section discussion. Chart generation: The data in all analytical tables (Tables SI-IV) are derived from original studies screened through this process, ensuring the evidence base of the table contents.

Through the aforementioned rigorous, transparent and structured literature search and screening process, the present review strived to build a comprehensive, balanced and timely evidence system to provide a reliable basis in elaborating on the transformation prospects of cfDNA in the full management of breast cancer.

Table SI. Summary of research on the pathogenesis and early diagnosis of breast cancer cfDNA gene-related markers.

| First author, year, | Research design[a] | Sample type | Sample capacity (case/control), n | Test method | Genes[b] | Key outcome measures | Evidence level and bias risk[c] | (Ref.) |
|---|---|---|---|---|---|---|---|---|
| Decker *et al*, 2017 | Case-control study | Blood and tissues | 13,087/5,488 | dPCR | *ATM*,*CHEK2*,*PALB2* and *XRCC2* | OR (95% CI): *ATM*, 2.78 (1.94-3.99); *CHEK2*, 2.20 (1.69-2.85); *PALB2*, 5.53 (2.64-11.58); *XRCC2*, NS | Class A (high reliability): Large-scale, multicenter case-control studies with notable sample sizes, high statistical power, well-controlled population stratification and low risk of bias | (141) |
| Li *et al*, 2018 | Case-control study | Blood plasma | 77/55 | BS-seq and MSP | *NBPF1* | AUC, 0.85; sensitivity, 79.2%; specificity, 80.0% | Class B (moderate reliability): Prospective single-center study with moderate sample size, providing diagnostic performance metrics, but requiring independent cohort validation | (142) |
| Shirkavand *et al*, 2018 | Case-control study | Whole blood | 60/40 | RT-qPCR | *VIM*,*CXCR4*, *DOK7* and *SPDEF* | Sensitivity/specificity range, 71.7-86.7%/72.5-92.5% | Class C (preliminary evidence): Small, single-center | (143) |

| | | | | | | | | |
|---|---|---|---|---|---|---|---|---|
| | | | | | | | exploratory study with limited sample size, providing preliminary performance data for multiple genes | |
| Douvdevani *et al*, 2020 | Cross-sectional study | Serum | 20/20 | qPCR | *BRCA1* and *BRCA2* | Median concentration of cfDNA: Mutant carriers vs. non-carriers, 1.32 vs. 0.72 ng/µl ($P<0.05$) | Class C (preliminary evidence): Small pilot study with a small sample size, designed to observe trends rather than confirm associations, with high risk of bias | (144) |
| Murillo Carrasco *et al*, 2021 | Case-control study | Blood plasma | 82/82 | dPCR | *PUM1* and *RPPH1* | AUC: *PUM1*, 0.73; *RPPH1*, 0.69; combination, 0.77 | Class B (moderate reliability): A case-control design with well-matched sample sizes, providing a quantitative assessment of diagnostic performance | (145) |
| Wang *et al*, 2021 | Case-control study | Plasma and tissue | 109/109 | qMSP | *GCM*, *ITPRIPL1* and *CCDC181* | AUC, 0.96/0.93; sensitivity for early breast cancer, 85.7% | Class B (moderate reliability): The study design is complete, | (146) |

| | | | | | | | | |
|---|---|---|---|---|---|---|---|---|
| | | | | | | | including training and validation sets, with exploratory approaches for both early diagnosis and efficacy monitoring and a moderate sample size | |
| Winter *et al*, 2022 | Cross-sectional study | Tissue and blood | 58/606 | Methylight PCR | *BCAT1* and *IKZF1* | Tissue testing rate, 86%; blood testing rate, 41% (in patients with breast cancer) | Category B (moderate reliability): Multicancer studies with relatively limited breast cancer sample size, focusing on the feasibility validation of cross-cancer technology applications | (147) |
| Danos *et al*, 2023 | Case-control study | Blood plasma | 58/58 | Methylight PCR | *RARB* and *GSTP1* | Sensitivity/specificity: *RARB*, 74.1/69.0%; *GSTP1*, 67.2/72.4%; combined, 81.0/65.5% | B-grade (moderate reliability): Single-center case-control study with moderate sample size, evaluated in a specific population | (148) |
| Grisolia *et al*, 2024 | Case-control | Blood plasma | 23/21 | cfMeDIP-seq | *BRCA1* and *BRCA2* | Diagnostic classification AUC, 0.91; *BRCA1/2* | Level C (preliminary | (149) |

| | | | | | | | | |
|---|---|---|---|---|---|---|---|---|
| | study design | | | | | mutation status prediction: High accuracy (specific value not specified in the text) | evidence): Proof-of-concept study with a small sample size, demonstrating the potential of high-throughput novel technologies, results require large-scale validation | |

[a]Research design: Case-control and cross-sectional; [b]Gene naming: All gene symbols are in italics and comply with the Human Gene Nomenclature Committee specifications; [c]Description of evidence level: i) Class A (high reliability): Large-scale, well-designed prospective or multicenter studies with low risk of bias; ii) class B (moderate reliability): Prospective single-center or well-designed retrospective study with moderate sample size and controllable risk of bias; and iii) level C (preliminary evidence): Small retrospective, exploratory or pilot study with limited sample size, the results need further verification and the risk of bias is relatively high. cfDNA, circulating cell-free DNA; OR, odds ratio; CI, confidence interval; AUC, area under the receiver operating characteristic curve; NS, not statistically significant; PCR, polymerase chain reaction; dPCR, digital PCR; BS-seq, bisulfite sequencing; MSP, methylation-specific PCR; RT-qPCR, reverse transcription-quantitative PCR; qMSP, quantitative MSP; cfMeDIP-seq, cell-free methylated DNA co-immunoprecipitation sequencing.

**Table SII.** Role of genetic testing in the evaluation and monitoring of treatment efficacy in breast cancer.

| First author, year | Research design[a] | Sample capacity | Sample type | Characteristics of breast cancer | Genes[b] | Test method | Evidence level and description[c] | Monitoring/therapeutic evaluation | (Ref.) |
|---|---|---|---|---|---|---|---|---|---|
| Takeshita *et al*, 2018 | Prospective cohort study | 128 | Blood plasma | $ER^+$, metastatic and primary | *PIK3CA*, *AKT1* and *ESR1* | ddPCR | Class B: Prospective design with moderate sample size, demonstrating the utility of ctDNA for dynamic monitoring | Disease surveillance | (172) |
| Kodahl *et al*, 2018 | Prospective cohort study | 66 | Serum and tumor tissue | Metastatic | *PIK3CA* | ddPCR | Class B: Prospective single-center study with moderate sample size and well-designed longitudinal monitoring | Disease surveillance | (173) |
| Winn *et al*, 2020 | Cross-sectional study | 19 | Tumor tissue and blood | Advanced inflammation | *TP53*, *PMS2*,*MRE11*, *RB1* and *BRCA1* | NGS | Class C: Small exploratory study with limited sample size, primarily aimed at verifying technical consistency | Disease surveillance | (174) |
| Zuo *et al*, 2020 | Case-control study | 250 | Plasma, urine and tumor tissue | Commitment | *PIK3CA* | ddPCR | Class B: Large sample size was used to explore a novel strategy of multi-fluid sample combined detection | Disease surveillance | (175) |

| | | | | | | | | | |
|---|---|---|---|---|---|---|---|---|---|
| Qui *et al*, 2021 | Cross-sectional study | 184 | Tumor tissue and blood | HER2$^{+}$, primary and metastatic | *HER2-CN* and*HER2-ECD* | ddPCR | Class B: Adequate sample size, with association between ctDNA and protein biomarkers | Disease surveillance | (176) |
| Sim *et al*, 2021 | Retrospective cohort study | 75 | Blood plasma | Metastatic and high-risk | *PIK3CA* and *ESR1* | | Class B: Retrospective design, moderate sample size, with clear clinical translation orientation | Treatment evaluation | (177) |
| Page *et al*, 2021 | Observational cohort study | 373 | Blood plasma | Primary metastatic | *PIK3CA*, *ESR1*and *TP53* | NGS | Class B: Large-sample cohort study providing a comprehensive description of ctDNA characteristics across disease stages | Disease surveillance | (178) |
| Keup *et al*, 2021 | Prospective longitudinal study | 27 | Blood | HR$^{+}$, HER2$^{-}$ and metastatic | *ESR1*, *PIK3CA*, *ERBB2*and *ERBB3* | qPCR and NGS | Class C: Conceptual validation study with a small sample size, but demonstrates the advancement of integrated multimodal liquid biopsy | Disease surveillance | (179) |
| Gerratana *et al*, 2023 | Retrospective cohort study | 703 | Blood | HR$^{+}$, HER2$^{-}$ and metastatic | *PIK3CA* and *ESR1* | NGS | Class A: Large-scale retrospective multicenter study with notable sample size, | Treatment evaluation | (180) |

| | | | | | | | | | |
|---|---|---|---|---|---|---|---|---|---|
| | | | | | | | further analysis and high statistical power | | |
| Martenes *et al*, 2024 | Retrospective/methodological study | 136 | Blood | Metastatic | *PIK3CA* | ddPCR | Class B: Methodologically oriented studies that provide a practical framework for the clinical interpretation of ctDNA quantitative results | Disease surveillance | (181) |
| Hattori *et al*, 2024 | Prospective cohort study | 78 | Blood and tumor tissue | $HR^+$, $HER2^-$ and metastatic | *PIK3CA*, *TP53*, *ESR1* and *GATA* | ddPCR | Class B: Prospective studies based on clinical trials, with homogeneous sample characteristics and strong association with treatment outcomes | Disease surveillance | (182) |

[a]Study design: Prospective cohort, retrospective cohort, cross-sectional, case-control and observational cohort; [b]Gene nomenclature: All human gene symbols are in italics, conforming to the standard; [c]Evidence level description (consistent with Table SI): i) Class A (high reliability): Large-scale, multicenter, well-designed studies; ii) category B (moderate reliability): Single-center prospective or well-designed large/medium-scale retrospective studies; and iii) level C (preliminary evidence): Small, exploratory, pilot or proof-of-concept studies. ctDNA, circulating tumor DNA; PCR, polymerase chain reaction; ddPCR, droplet digital PCR; NGS, next-generation sequencing; qPCR, quantitative PCR; ER, estrogen receptor; HR, hormone receptor; HER2, human epidermal growth factor receptor 2; CN, copy number; ECD, extracellular domain.

**Table SIII.** Association study of gene mutations with prognosis in patients with breast cancer.

| First author, year | Research design[a] | Sample capacity | Sample type | Genes[b] | Test method | Characteristics of breast cancer | Key prognostic outcomes | Evidence level and description[c] | (Ref.) |
|---|---|---|---|---|---|---|---|---|---|
| Chandarlapaty *et al*, 2016 | Secondary analysis of clinical trials | 541 | Blood plasma | *ESR1* | ddPCR | Metastatic | OS: Patients with *ESR1* mutation had significantly shorter OS (HR and P-values reported in the original text); mutation rate, 28.8% | Class A: High-quality evidence from prospective samples based on large randomized controlled trials, with low risk of bias | (215) |
| Li *et al*, 2017 | Retrospective case-control study | 84 | Serum and tumor tissue | *PVT1* | RT-qPCR | Invasiveness of the catheter | 5-year OS: OS was significantly shortened in patients with high serum *PVT1* expression ($P<0.05$) | Class C: Small single-center exploratory study with limited sample size, first reported the prognostic value of PVT1 in serum, requiring validation | (216) |
| Jacot *et al*, 2019 | Prospective longitudinal cohort study | 39 | Blood plasma | *PIK3CA* | ddPCR | Metastatic | PFS: The presence of *PIK3CA* mutations in ctDNA 4 weeks after treatment was strongly associated with shorter PFS (HR value as reported in the original study) | Class C: The prospective design is notable, but the sample size is small and the results are generated from notable hypotheses | (217) |

| | | | | | | | | | |
|---|---|---|---|---|---|---|---|---|---|
| Keup *et al*, 2020 | Prospective longitudinal cohort study | 44 | Blood plasma | *AKT1*, *AR*,*BRCA1*, *BRCA2*, *MUC16* and *ERBB3* | NGS | $ER^+$ and $PR^+$ metastatic | OS: Patients with specific *BRCA1* mutations and ≥3.5 pathogenic mutations had shorter OS; longitudinal monitoring revealed increased mutation burden at the time of drug resistance | Class C: Small longitudinal study with limited sample size, involving polygenic exploration | (218) |
| Muendlein *et al*, 2021 | Prospective cohort study | 59 | Blood plasma | *ESR1*,*PIK3CA*,*ERBB2*,*PTEN*,*TP53*, *KRAS*, *HRAS*, *NRAS* and*AR* | NGS | $ER^+$, $HER2^-$ and metastatic | PFS and OS: The number of detected mutations in ctDNA is associated with worse PFS and OS; mutational status of *ESR1* and *TP53* serves as predictors of PFS and OS | Category C: Single-center prospective study with small sample size, demonstrating clear results but requiring expanded validation | (219) |
| Liao *et al*, 2022 | Retrospective cohort study | 141 | Blood plasma | *ERBB2*, *FGFR1*,*CDKN2A*, *TP53* and *PIK3CA* | NGS | Senior | OS and PFS: In TNBC, *TP53* or *PIK3CA* mutations are associated with shorter OS; a high ctDNA score (composite marker) is associated with shorter PFS and OS across all subtypes | Category B: Moderate sample size, covering various subtypes with comprehensive analysis | (220) |

| | | | | | | | | | |
|---|---|---|---|---|---|---|---|---|---|
| Zhang *et al*, 2022 | Retrospective cohort study | 101 | Tumor tissue and plasma | *TP53*, *CTCF*, *GNAS* and *Notch1* | NGS | Recurrent and metastatic | OS: *TP53*, CTCF and Notch1 mutations are associated with poor prognosis; newly detected *TP53* mutations predict worse OS | Class B: Medium sample size, with analysis of both tissue and plasma samples, focusing on the prognostic value of specific genes | (221) |
| Tolaney *et al*, 2022 | Secondary analysis of clinical trials | 669 | Blood plasma | *PIK3CA* and *ESR1* | ddPCR | $HR^+$ and HER2-advanced | PFS and OS: In the abemaciclib + fulvestrant treatment group, patients with *PIK3CA* or *ESR1*ctDNA mutations had longer PFS and OS (treatment interaction) | Class A: Prognostic biomarker analysis based on large-scale Phase III RCTs, with the highest evidence strength, directly guiding treatment | (222) |
| Chen *et al*, 2023 | Retrospective cohort study | 163 | Blood plasma | *PIK3CA* and *TP53* | NGS | $ER^+$ and HER2-metastatic | PFS: High cfDNA concentration, *PIK3CA* and *TP53*mutations were associated with worse PFS (HR values reported in the original study) | Category B: Moderate sample size, retrospective design, with simultaneous evaluation of concentration and specific gene mutations | (223) |

| | | | | | | | | | |
|---|---|---|---|---|---|---|---|---|---|
| Dickinson *et al*, 2024 | Systematic reviews and meta-analyses | 4,264 | Blood plasma | *TP53*, *ESR1* and *PIK3CA* | NGS and ddPCR | Metastatic stage IV | OS: Meta-analysis demonstrated that ctDNA detection was associated with worse OS; *TP53* and *ESR1* mutations exhibited significant negative prognostic value, whereas *PIK3CA* mutations indicated no such association in this analysis | Grade A: Synthesis of high-level evidence with a large sample size, robust conclusions, representing the highest level of evidence currently available | (224) |
| Takeshita *et al*, 2024 | Retrospective study | 65 | Blood plasma | *ESR1*, *PIK3CA*, *FOXA1*, *RUNX1*, *TBX3* and *TP53* | NGS | $HR^+$, $HER2^-$, advanced and metastatic | PFS: Baseline *PIK3CA* mutation was associated with worse PFS; the prognostic relevance of *TP53* and *PIK3CA* mutations was enhanced after treatment | Class B: Medium sample size, exploring novel biomarkers in patients with fluvistatin resistance | (225) |

[a]Study design: Randomized controlled trial, prospective cohort, retrospective cohort, systematic review/metastatic analysis and case-control; [b]Gene nomenclature: All human gene symbols are in italics, conforming to the standard; [c]Evidence level description (consistent with Table I and II): i) Class A (high reliability): Prespecified or prospective biomarker analysis in large randomized controlled trials, large-scale prospective multicenter cohorts, high-quality systematic reviews/metastatic analyses; ii) category B (moderate reliability): Single-center prospective cohort studies or moderately to large retrospective cohort studies; and iii) class C (preliminary evidence): Small exploratory, pilot and case-control studies. ctDNA, circulating tumor DNA; HR, hazard ratio, used for survival analysis; OS, overall survival; PFS, progression-free survival; ddPCR, droplet digital PCR; NGS, next generation sequencing; RT-qPCR, reverse-transcription fluorescent quantitative PCR; ER, estrogen receptor; PR, progesterone receptor; HER2, human epidermal growth factor receptor 2; TNBC, triple-negative breast cancer; RCT, randomized controlled trial; AR, androgen receptor.

**Table SIV.** Summary of evidence on the prognostic value of key gene mutations in breast cancer cfDNA.

| Genes[a] | Number of studies | Total number of patients (approximate count) | Research design | Relevant survival rate indicators | Research conclusion | Summary of prognostic association conclusions[b] | Consistency of evidence[c] | (Refs.) |
|---|---|---|---|---|---|---|---|---|
| *TP53* | 6 | >4,600 | Prospective/retrospective cohort and systematic review | OS, PFS and DFS | Markedly associated with reduced survival rate | Consistent findings indicate poor prognosis; detection of *TP53* mutations in ctDNA is markedly associated with shorter OS, PFS and DFS; systematic reviews have confirmed its strong prognostic value | Evidence is robust and consistent; multiple independent studies on metastatic and advanced breast cancer have reached consistent conclusions, supported by large-sample systematic reviews | (219-221,223-225) |
| *ESR1* | 6 | >1,300 | RCT secondary analysis, prospective/review cohort and systematic review | OS, PFS and DFS | Markedly associated with reduced survival rate | A clear indication of poor prognosis, particularly associated with resistance to endocrine therapy; in metastatic $HR^+$ breast cancer, *ESR1* mutation serves as an independent negative predictor of OS and PFS; studies | Evidence is robust; based on high-quality clinical trial data and large-scale real-world studies, the conclusion is robust; it serves as a key biomarker for $HR^+$ breast cancer prognosis and resistance monitoring | (215,218,219,222,224,225) |

| | | | | | | | | |
|---|---|---|---|---|---|---|---|---|
| | | | | | | suggest that its significance may vary under specific treatment regimens | | |
| *PIK3CA* | 8 | >1,500 | Prospective/re retrospective cohort studies, secondary analysis of RCTs and systematic reviews | OS, PFS, RFS and BCSS | Markedly associated with reduced survival rate | Most evidence suggests a poor prognosis, but there is heterogeneity; multiple studies have demonstrated its association with shorter PFS and OS; however, large systematic reviews did not find a significant association with OS and in certain specific treatment contexts (for example, combination therapy with CDK4/6 inhibitors), the association may be attenuated or of different significance | Evidence is of moderate strength and requires interpretation in conjunction with clinical context; it is generally considered an adverse prognostic factor, but its independent prognostic value may be influenced by treatment modality, mutation status and co-mutations | (217-220,222-225) |
| *AR* | 3 | <126 | Prospective/retrospective cohort | PFS | Markedly associated with reduced | Preliminary findings suggest an unfavorable | Evidence is preliminary and requires | (218,219,225) |

| | | | | | | | | |
|---|---|---|---|---|---|---|---|---|
| | | | | | survival rate | prognosis; in limited studies, *AR* mutations in ctDNA are associated with shorter PFS | validation; the conclusion is based on exploratory studies with small sample sizes and needs to be confirmed in larger cohorts for its prognostic value in specific subtypes (for example, $AR^+$ breast cancer) | |
| *PVT1* | 1 | 84 | Retrospective case control | OS and DFS | Associated with poor OS | Preliminary findings suggest poor prognosis; high expression of *PVT1* in serum is associated with worse 5-year OS and DFS | Evidence is preliminary; this is a single-center, small-sample study and its reproducibility needs to be validated in ctDNA or an independent cohort | (216) |
| *CTCF* and *GNAS* | 1 | 101 | Retrospective cohort | DFS | Associated with worse DFS | Preliminary findings suggest poor prognosis; associated with worse DFS | Evidence is preliminary; single study result requires independent validation | (221) |
| *Notch1* | 1 | 101 | Retrospective cohort | OS | Associated with poor OS | Preliminary indication of poor prognosis; | Evidence is preliminary; the single study | (221) |

| | | | | | | | | |
|---|---|---|---|---|---|---|---|---|
| | | | | | | associated with worse OS | result requires independent validation | |

[a]Gene nomenclature: All gene symbols are in italics;[b]Prognostic association direction: This mutation is associated with worse survival; [c]Explanation of evidence strength assessment: i) Conclusion: The findings are supported by large-sample prospective cohorts, high-quality secondary analyses of RCTs or systematic reviews/meta-analyses, with consistent conclusions across studies; ii) moderate: The conclusion is supported by multiple studies, but the sample size is moderate or exhibits certain heterogeneity or requires interpretation in conjunction with clinical context; and iii) preliminary findings: The conclusions are based solely on single-item or small-sample studies, requiring urgent independent validation. ctDNA, circulating tumor DNA; OS, overall survival PFS, progression-free survival; DFS, disease-free survival; RFS, relapse-free survival; BCSS, breast cancer-specific survival; RCT, randomized controlled trial; HR, hormone receptor; AR, androgen receptor.
